# Supplementary figures and images for: Investigation of the quorum-sensing regulon of the biocontrol bacterium Pseudomonas chlororaphis strain PA23
Source: PLoS One. 2020 Feb 28;15(2):e0226232. doi: 10.1371/journal.pone.0226232 (PMC7048289; doi:10.1371/journal.pone.0226232)

# Supplemental Figure 1

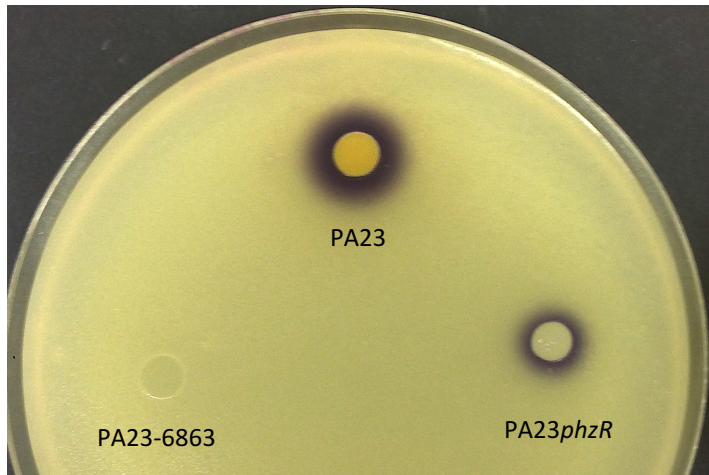

Supplement: S1 Fig — Picture is representative of five biological replicates obtained from three independent experiments. (PDF) [file pone.0226232.s001.pdf]

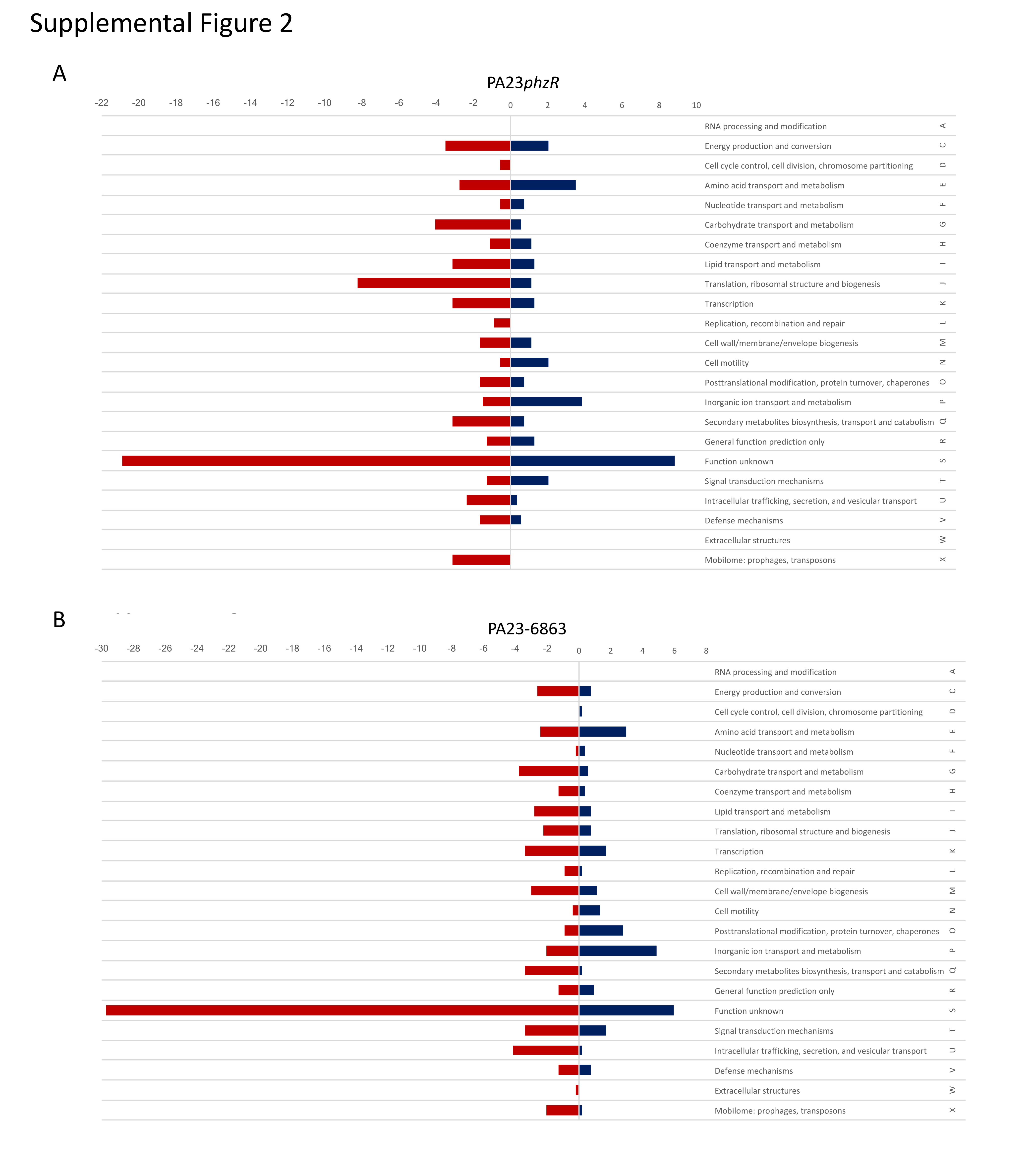

Supplement: S2 Fig — Functional analysis of differentially expressed genes in (A) PA23phzR and (B) PA23-6863 in comparison to wild type using Cluster of Orthologous Group (COG) analysis. The red bars indicate percent of differentially regulated genes that are downregulated, and the blue bars indicate percentage of genes that are upregulated in each category. (TIFF) [file pone.0226232.s002.tiff]

# Supplemental Figure 3

A

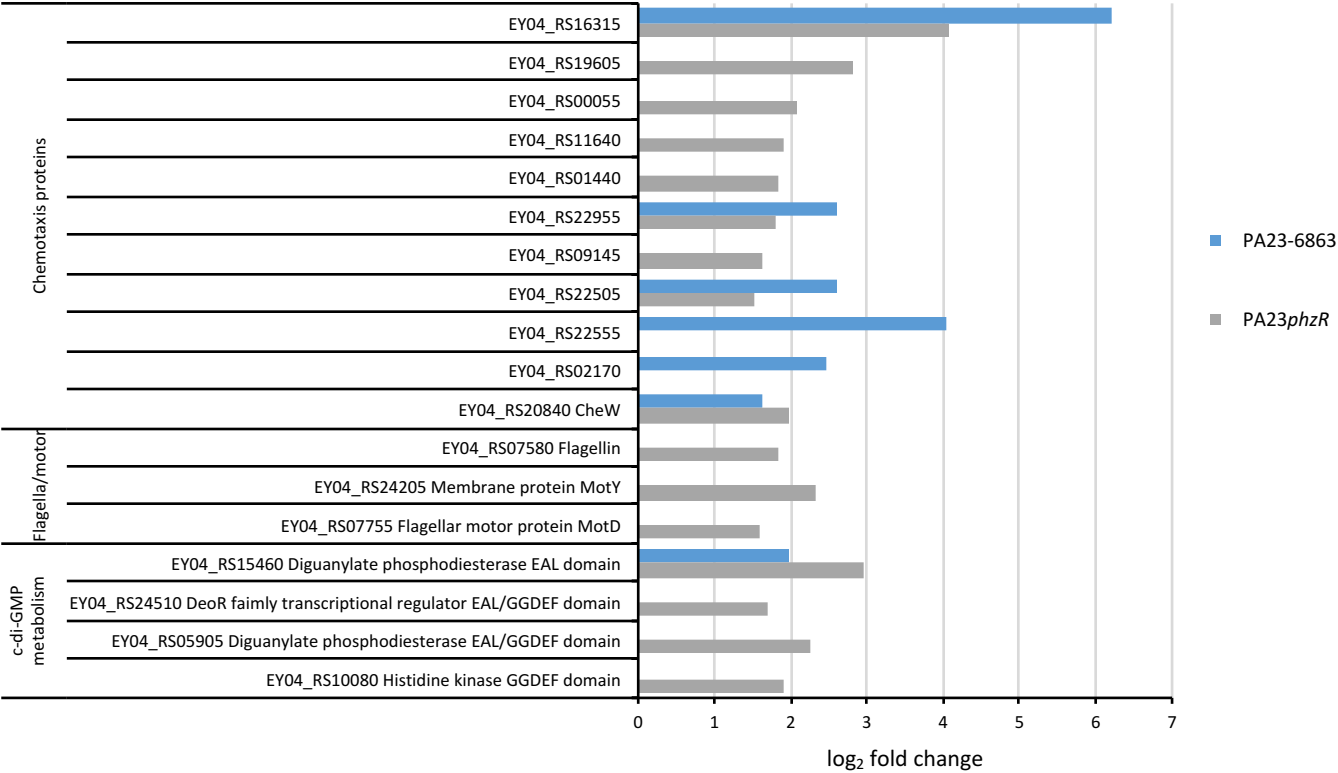

B

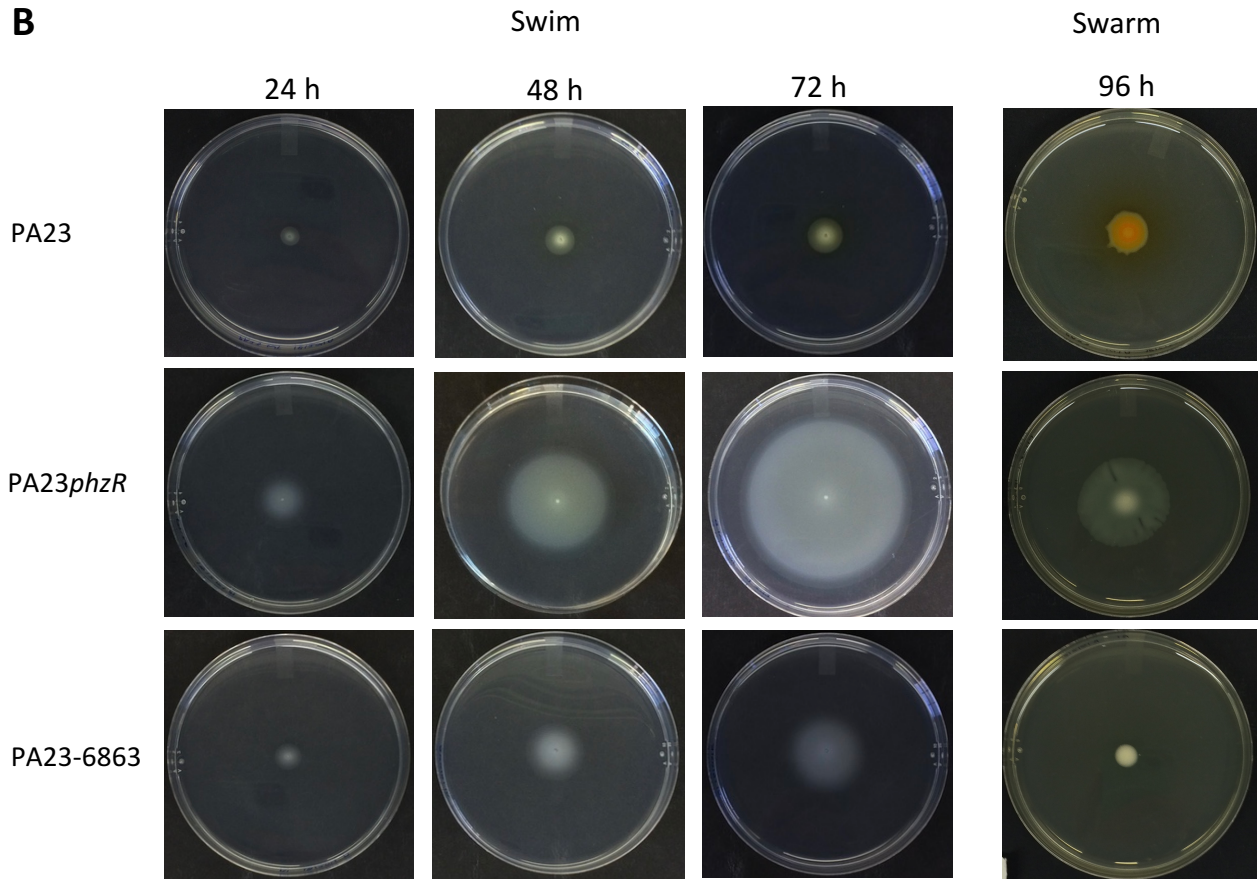

Supplement: S3 Fig — A) Motility genes upregulated in PA23phzR and PA23-6863 compared to PA23. B) Swim plates (0.3% agar) after 24, 48, and 72 h of incubation; Swarm plates (0.8% agar) after 96 h incubation. Pictures are representative of five biological replicates obtained from three independent experiments. (PDF) [file pone.0226232.s003.pdf]
