# Supplementary material for: Investigation of the quorum-sensing regulon of the biocontrol bacterium Pseudomonas chlororaphis strain PA23
Source: PLoS One. 2020 Feb 28;15(2):e0226232. doi: 10.1371/journal.pone.0226232 (PMC7048289; doi:10.1371/journal.pone.0226232)
Supplement: S1 Table — (DOCX) [file pone.0226232.s004.docx]

**Table S1.** RNA sequencing library reads mapped to the *Pseudomonas chlororaphis* PA23 genome

| **Strain** | **Total reads** | **Reads mapped to PA23 genome** | **PA23 mapping %** |
| --- | --- | --- | --- |
| **(Replicate)** |  |  |  |
| **PA23 WT (1)** | 4,662,579 | 4,354,719 | 93.4 |
| **PA23 WT (2)** | 4,722,434 | 4,504,607 | 95.4 |
| **PA23 WT (3)** | 4,485,424 | 4,271,368 | 95.2 |
| **PA23-6863 (1)** | 5,940,843 | 5,676,126 | 95.5 |
| **PA23-6863 (2)** | 2,940,079 | 2,860,126 | 97.3 |
| **PA23-6863 (3)** | 9,850,960 | 9,447,090 | 95.9 |
| **PA23*phzR* (1)** | 6,569,876 | 6,295,963 | 95.8 |
| **PA23*phzR* (2)** | 9,330,419 | 8,921,174 | 95.6 |
| **PA23*phzR* (3)** | 7,937,214 | 7,667,498 | 96.6 |
