# Supplementary material for: Investigation of the quorum-sensing regulon of the biocontrol bacterium Pseudomonas chlororaphis strain PA23
Source: PLoS One. 2020 Feb 28;15(2):e0226232. doi: 10.1371/journal.pone.0226232 (PMC7048289; doi:10.1371/journal.pone.0226232)
Supplement: S2 Table — (DOCX) [file pone.0226232.s005.docx]

**Table S2.** Differentially expressed genes in PA23*phzR* relative to PA23 wild type

| Functional category | Predicted function | Locus tag | COG accession | COG category | Log2 fold change | Adjusted P-value |
| --- | --- | --- | --- | --- | --- | --- |
| Energy production and conversion | Aldehyde dehydrogenase | EY04_RS10625 | COG1012 | C | 4.69 | 9.10E-04 |
|  | Branched-chain alpha-keto acid dehydrogenase subunit E2 | EY04_RS11260 | COG0508 | C | 2.81 | 9.60E-06 |
|  | Cytochrome C | EY04_RS16565 | cl21467 | - | 2.57 | 9.09E-03 |
|  | NADH dehydrogenase | EY04_RS19100 | COG0377 | C | 2.04 | 2.06E-11 |
|  | Dihydrolipoamide dehydrogenase | EY04_RS11255 | COG1249 | C | 2.04 | 7.82E-04 |
|  | 2-hydroxyacid dehydrogenase | EY04_RS11815 | COG1052 | CHR | 1.78 | 1.01E-03 |
|  | 2-oxoisovalerate dehydrogenase | EY04_RS11265 | COG0022 | C | 1.72 | 3.08E-03 |
|  | Alcohol dehydrogenase | EY04_RS16600 | COG0604 | CR | 1.71 | 1.19E-03 |
|  | Succinate-semialdehyde dehydrogenase | EY04_RS00410 | COG1012 | C | 1.67 | 3.59E-11 |
|  | 3-isopropylmalate dehydrogenase | EY04_RS09545 | COG0473 | CE | 1.61 | 4.24E-03 |
|  | ACP phosphodiesterase | EY04_RS12795 | COG0431 | C | 1.60 | 4.19E-03 |
|  | Succinyl-CoA synthetase subunit beta | EY04_RS08050 | COG0045 | C | -1.66 | 7.72E-14 |
|  | Malate dehydrogenase | EY04_RS01565 | COG0281 | C | -1.70 | 2.08E-07 |
|  | MFS transporter | EY04_RS16325 | COG1853 | C | -1.70 | 8.52E-06 |
|  | Dihydrolipoamide succinyltransferase | EY04_RS08040 | COG0508 | C | -1.72 | 2.82E-13 |
|  | ATP F0F1 synthase subunit alpha | EY04_RS28925 | COG0056 | C | -1.73 | 1.58E-21 |
|  | Dihydrolipoamide acetyltransferase | EY04_RS01835 | COG0508 | C | -1.74 | 1.08E-17 |
|  | Cytochrome D ubiquinol oxidase subunit III | EY04_RS25240 | cl11473 | - | -1.80 | 4.70E-07 |
|  | Pyruvate dehydrogenase | EY04_RS01840 | COG2609 | C | -1.81 | 8.77E-13 |
|  | 2-oxoglutarate dehydrogenase | EY04_RS08035 | COG0567 | C | -1.81 | 5.32E-18 |
|  | ATP synthase F0F1 subunit delta | EY04_RS28920 | COG0712 | C | -1.88 | 6.34E-18 |
|  | Succinate dehydrogenase | EY04_RS08020 | COG2142 | C | -1.92 | 1.02E-19 |
|  | 2Fe-2S ferredoxin | EY04_RS24695 | COG0633 | C | -1.97 | 4.63E-05 |
|  | Iron ABC transporter substrate-binding protein | EY04_RS00885 | COG1526 | C | -1.99 | 1.75E-04 |
|  | Alcohol dehydrogenase | EY04_RS28585 | COG2010 | C | -2.02 | 1.95E-03 |
|  | CbbBc protein | EY04_RS00880 | COG0243 | C | -2.11 | 3.87E-07 |
|  | Ferredoxin-NADP reductase | EY04_RS05580 | COG1018 | C | -2.11 | 5.88E-19 |
|  | Glycerol kinase | EY04_RS24270 | COG0554 | C | -2.14 | 4.58E-13 |
|  | (Fe-S)-binding protein | EY04_RS13285 | COG0348 | C | -3.80 | 5.95E-23 |
|  | cbb3-type cytochrome c oxidase subunit I | EY04_RS13215 | COG3278 | C | -5.36 | 1.97E-76 |
| Cell cycle control, cell division, chromosome partitioning | GTPase CgtA | EY04_RS26500 | COG0536 | DL | -1.60 | 6.41E-06 |
|  | Cell division protein FtsZ | EY04_RS25075 | COG0206 | D | -1.74 | 7.40E-10 |
|  | Cell division topological specificity factor | EY04_RS21725 | COG0851 | D | -1.74 | 2.88E-06 |
| Amino acid transport and metabolism | Phosphoribosyl-AMP cyclohydrolase | EY04_RS29145 | COG0139 | E | 6.40 | 1.46E-06 |
|  | Allantoate amidohydrolase | EY04_RS20520 | COG0624 | E | 5.29 | 2.11E-04 |
|  | Diaminobutyrate--2-oxoglutarate aminotransferase | EY04_RS15405 | COG0160 | E | 4.12 | 2.43E-13 |
|  | ABC transporter ATP-binding protein | EY04_RS05865 | COG0410 | E | 3.63 | 4.86E-03 |
|  | Gamma-glutamyl-gamma-aminobutyrate hydrolase | EY04_RS10615 | COG2071 | E | 3.25 | 1.94E-03 |
|  | Allantoate amidohydrolase | EY04_RS11340 | COG0624 | E | 3.18 | 8.80E-04 |
|  | Spermidine/putrescine ABC transporter permease | EY04_RS30395 | COG1177 | E | 2.80 | 1.28E-04 |
|  | FAD-dependent oxidoreductase | EY04_RS15450 | COG0665 | E | 2.60 | 9.42E-09 |
|  | 3-isopropylmalate dehydratase | EY04_RS09540 | COG0066 | E | 2.47 | 1.27E-03 |
|  | 4-aminobutyrate aminotransferase | EY04_RS00415 | COG0160 | E | 2.29 | 8.55E-10 |
|  | Gamma-glutamylputrescine synthetase | EY04_RS30355 | COG0174 | E | 2.14 | 3.15E-15 |
|  | Gamma-glutamylputrescine synthetase | EY04_RS10620 | COG0174 | E | 2.12 | 1.65E-03 |
|  | Isopropylmalate isomerase | EY04_RS09535 | COG0065 | E | 2.06 | 1.42E-03 |
|  | Argininosuccinate lyase | EY04_RS14115 | COG0165 | E | 1.80 | 1.60E-03 |
|  | Glycine cleavage system protein R | EY04_RS31420 | COG2716 | E | 1.62 | 5.84E-03 |
|  | Ornithine acetyltransferase | EY04_RS23815 | COG1364 | E | 1.55 | 7.75E-03 |
|  | Gamma-aminobutyrate transporter | EY04_RS01120 | COG1113 | E | 1.53 | 3.80E-03 |
|  | Amino acid ABC transporter substrate-binding protein | EY04_RS04525 | COG0834 | ET | 1.52 | 5.23E-04 |
|  | Gamma-glutamyl kinase | EY04_RS26495 | COG0263 | E | 1.52 | 3.18E-04 |
|  | Methionine synthase | EY04_RS18470 | COG1410 | E | -1.53 | 4.69E-09 |
|  | Amino acid ABC transporter substrate-binding protein | EY04_RS30955 | COG0834 | ET | -1.61 | 1.10E-03 |
|  | 2,3,4,5-tetrahydropyridine-2,6-carboxylate N-succinyltransferase | EY04_RS05120 | COG2171 | E | -1.62 | 2.08E-06 |
|  | Glutamine synthetase | EY04_RS01280 | COG0174 | E | -1.68 | 6.27E-13 |
|  | Glycine cleavage system protein T | EY04_RS30240 | COG0404 | E | -1.69 | 3.34E-09 |
|  | 5-methyltetrahydropteroyltriglutamate-- homocysteine methyltransferase | EY04_RS24380 | COG0620 | E | -1.81 | 6.91E-03 |
|  | Hypothetical protein | EY04_RS24300 | COG1126 | E | -1.85 | 1.59E-09 |
|  | Glutamate-pyruvate aminotransferase | EY04_RS22695 | COG0436 | E | -1.90 | 2.31E-09 |
|  | Urocanate hydratase | EY04_RS01355 | COG2987 | E | -1.91 | 2.38E-06 |
|  | ABC transporter substrate-binding protein | EY04_RS14255 | COG0834 | ET | -2.21 | 1.33E-03 |
|  | ABC transporter | EY04_RS24285 | COG0834 | ET | -2.32 | 9.61E-19 |
|  | Phosphogluconate dehydratase | EY04_RS22915 | COG0129 | EG | -2.91 | 1.97E-19 |
|  | Hydrogen cyanide synthase HcnC | EY04_RS11550 | COG0665 | E | -3.35 | 5.88E-28 |
|  | Diaminopimelate decarboxylase | EY04_RS10485 | COG0019 | E | -3.36 | 1.11E-10 |
|  | Serine/threonine protein kinase | EY04_RS11845 | COG0814 | E | -3.62 | 3.98E-24 |
| Nucleotide transport and metabolism | Dihydroorotase | EY04_RS29135 | COG0044 | F | 7.32 | 2.66E-08 |
|  | Nitrate reductase | EY04_RS11335 | COG1953 | FH | 2.27 | 2.91E-03 |
|  | Xanthine dehydrogenase | EY04_RS08950 | COG4631 | F | 2.23 | 2.08E-05 |
|  | Amidotransferase | EY04_RS08680 | COG0518 | F | 1.59 | 1.56E-05 |
|  | Ribose-phosphate pyrophosphokinase | EY04_RS25570 | COG0462 | FE | -1.64 | 5.53E-12 |
|  | Inosine-5-monophosphate dehydrogenase | EY04_RS24595 | COG0516 | F | -1.67 | 3.07E-13 |
|  | Adenylosuccinate lyase | EY04_RS19070 | COG0015 | F | -1.82 | 1.38E-05 |
| Carbohydrate transport and metabolism | MFS transporter | EY04_RS15390 | COG2814 | G | 4.89 | 1.40E-04 |
|  | LysR family transcriptional regulator | EY04_RS20525 | COG0477 | GEPR | 3.55 | 8.13E-03 |
|  | 4-alpha-glucanotransferase | EY04_RS13365 | COG1640 | G | 1.59 | 2.77E-03 |
|  | Sorbosone dehydrogenase | EY04_RS22005 | COG2133 | G | -1.65 | 2.12E-09 |
|  | Glycerol uptake facilitator GlpF | EY04_RS24265 | COG0580 | G | -1.94 | 6.36E-09 |
|  | Multidrug resistance protein B | EY04_RS19225 | COG2814 | G | -2.62 | 7.44E-10 |
|  | sn-glycerol-3-phosphate transporter | EY04_RS30160 | COG2271 | G | -2.86 | 6.23E-13 |
|  | DSBA oxidoreductase | EY04_RS00150 | COG2814 | G | -3.12 | 4.07E-06 |
|  | Sugar ABC transporter permease | EY04_RS22880 | COG0395 | G | -3.13 | 1.05E-08 |
|  | Permease DsdX | EY04_RS22700 | COG2610 | GR | -3.38 | 7.97E-16 |
|  | Gluconokinase | EY04_RS22705 | COG3265 | G | -3.52 | 2.03E-09 |
|  | Sugar ABC transporter ATPase | EY04_RS22875 | COG3839 | G | -3.63 | 1.90E-19 |
|  | Glucose-6-phosphate dehydrogenase | EY04_RS22855 | COG0364 | G | -3.80 | 1.86E-34 |
|  | 6-phosphogluconolactonase | EY04_RS22850 | COG0363 | G | -3.90 | 3.21E-43 |
|  | Methylglyoxal synthase | EY04_RS22925 | COG1803 | G | -3.91 | 2.22E-15 |
|  | MFS transporter | EY04_RS23950 | COG2814 | G | -4.02 | 7.91E-15 |
|  | Sugar ABC transporter permease | EY04_RS22885 | COG1175 | G | -4.31 | 5.30E-13 |
|  | Keto-deoxy-phosphogluconate aldolase | EY04_RS22845 | COG0800 | G | -4.38 | 1.19E-73 |
|  | Glyceraldehyde-3-phosphate dehydrogenase | EY04_RS22920 | COG0057 | G | -4.91 | 1.26E-85 |
|  | Chitin-binding protein | EY04_RS16025 | cl27306 | - | -5.38 | 7.11E-71 |
|  | Sugar ABC transporter substrate-binding protein | EY04_RS22890 | COG1653 | G | -5.58 | 2.60E-28 |
|  | Transporter | EY04_RS10775 | COG0697 | GER | -6.50 | 1.37E-45 |
|  | Chitinase | EY04_RS16020 | COG3469 | G | -6.91 | 9.05E-122 |
|  | Chitin-binding protein | EY04_RS09700 | cl27306 | - | -8.06 | 4.71E-289 |
|  | Chitinase | EY04_RS09705 | COG3469 | G | -8.51 | 0.00E+00 |
| Coenzyme transport and metabolism | Molybdenum cofactor biosynthesis protein MoaC | EY04_RS04480 | COG0315 | H | 2.83 | 1.68E-06 |
|  | Molybdenum cofactor biosynthesis protein MoaE | EY04_RS04490 | COG0314 | H | 2.30 | 8.12E-03 |
|  | Lipoate--protein ligase | EY04_RS27530 | COG0321 | H | 1.71 | 3.69E-04 |
|  | Cobalamin biosynthesis protein CobN | EY04_RS18430 | COG1429 | H | 1.61 | 1.70E-04 |
|  | Radical SAM protein | EY04_RS08500 | COG2896 | H | 1.60 | 5.10E-06 |
|  | Sirohydrochlorin ferrochelatase | EY04_RS18980 | COG0007 | H | 1.57 | 2.61E-04 |
|  | Bifunctional pyrazinamidase/nicotinamidase | EY04_RS16235 | COG1335 | HR | -1.69 | 3.30E-14 |
|  | 4-hydroxybenzoate polyprenyltransferase | EY04_RS29460 | COG0382 | H | -1.75 | 2.72E-03 |
|  | Omega amino acid--pyruvate aminotransferase | EY04_RS02980 | COG0161 | H | -1.91 | 8.48E-11 |
|  | Adenosylhomocysteinase | EY04_RS30970 | COG0499 | H | -1.97 | 4.66E-21 |
|  | Hypothetical protein | EY04_RS29920 | COG0432 | H | -2.32 | 2.88E-10 |
|  | MFS transporter | EY04_RS06330 | COG0596 | HR | -3.95 | 1.46E-71 |
| Lipid transport and metabolism | 3-hydroxy-2-methylbutyryl-CoA dehydrogenase | EY04_RS14040 | COG1028 | IQR | 5.86 | 2.20E-05 |
|  | AMP-binding protein | EY04_RS14035 | COG0365 | I | 5.21 | 1.06E-05 |
|  | Diaminopimelate decarboxylase | EY04_RS15395 | cl27723 | - | 4.66 | 2.23E-04 |
|  | Methylcrotonoyl-CoA carboxylase | EY04_RS19305 | COG4799 | I | 3.48 | 8.77E-04 |
|  | Choline dehydrogenase | EY04_RS11095 | COG2303 | IR | 3.24 | 6.39E-03 |
|  | Isovaleryl-CoA dehydrogenase | EY04_RS19300 | COG1960 | I | 2.29 | 6.91E-03 |
|  | Acetyl-CoA acetyltransferase | EY04_RS14045 | COG0183 | I | 1.82 | 1.69E-03 |
|  | Kinase | EY04_RS25560 | COG1947 | I | -1.58 | 4.88E-10 |
|  | Enoyl-CoA hydratase | EY04_RS08695 | COG1024 | I | -1.59 | 6.69E-04 |
|  | Glycerol acyltransferase | EY04_RS28810 | COG0204 | I | -1.64 | 2.74E-07 |
|  | Poly(R)-hydroxyalkanoic acid synthase | EY04_RS01520 | COG3243 | I | -1.89 | 1.65E-17 |
|  | Malonyl CoA-ACP transacylase | EY04_RS08445 | COG0331 | I | -2.00 | 8.16E-09 |
|  | Fatty acid methyltransferase | EY04_RS29115 | COG2230 | I | -2.04 | 8.37E-13 |
|  | Long-chain fatty acid--CoA ligase | EY04_RS22795 | COG0318 | IQ | -2.10 | 1.53E-17 |
|  | 3-ketoacyl-ACP reductase | EY04_RS08450 | COG1028 | IQR | -2.13 | 3.34E-15 |
|  | Acetyl-CoA carboxylase subunit alpha | EY04_RS05230 | COG0825 | I | -2.18 | 7.45E-19 |
|  | Trans-2-enoyl-CoA reductase | EY04_RS12495 | COG3007 | I | -2.21 | 1.10E-10 |
|  | GMC family oxidoreductase | EY04_RS28590 | COG2303 | IR | -2.24 | 5.34E-03 |
|  | Phosphate acyltransferase | EY04_RS08440 | COG0416 | I | -2.43 | 6.60E-14 |
|  | 3-oxoacyl-ACP synthase | EY04_RS09880 | COG0304 | IQ | -2.48 | 7.80E-07 |
|  | Poly(3-hydroxyalkanoate) granule-associated protein PhaF | EY04_RS01500 | - | - | -2.57 | 2.28E-34 |
|  | Glycerol acyltransferase | EY04_RS21875 | COG0204 | I | -2.93 | 1.15E-25 |
|  | (2Fe-2S)-binding protein HcnB | EY04_RS11545 | COG0446 | I | -3.47 | 1.72E-36 |
|  | Poly(3-hydroxyalkanoate) granule-associated protein PhaI | EY04_RS01495 | - | - | -4.50 | 1.50E-163 |
| Translation, ribosomal structure and biogenesis | Molecular chaperone DnaK | EY04_RS29180 | COG1734 | J | 8.16 | 4.31E-10 |
|  | Threonyl-tRNA synthetase | EY04_RS29130 | COG0441 | J | 5.46 | 3.92E-06 |
|  | 50S ribosomal protein L31 | EY04_RS24405 | COG0254 | J | 3.62 | 5.03E-05 |
|  | Alanyl-tRNA synthetase | EY04_RS08305 | cl26819 | - | 1.66 | 1.09E-07 |
|  | Ribosome hibernation promoting factor HPF | EY04_RS03925 | COG1544 | J | 1.60 | 8.31E-06 |
|  | Hypothetical protein | EY04_RS07390 | COG0009 | J | 1.57 | 3.10E-03 |
|  | Glucose-1-phosphate cytidylyltransferase | EY04_RS07500 | COG1208 | JM | -1.51 | 3.69E-08 |
|  | Tyrosine--tRNA ligase | EY04_RS31840 | COG0162 | J | -1.57 | 2.49E-06 |
|  | 30S ribosomal protein S16 | EY04_RS04765 | COG0228 | J | -1.69 | 3.19E-13 |
|  | tRNA (guanine-N1)-methyltransferase | EY04_RS04775 | COG0336 | J | -1.70 | 1.67E-10 |
|  | 50S ribosomal protein L19 | EY04_RS04780 | COG0335 | J | -1.81 | 9.94E-12 |
|  | GTP-binding protein | EY04_RS14290 | COG0480 | J | -1.81 | 7.84E-10 |
|  | Thiamine biosynthesis protein ThiF | EY04_RS05105 | COG1179 | J | -1.82 | 1.91E-03 |
|  | 50S ribosomal protein L25 | EY04_RS25575 | COG1825 | J | -1.89 | 2.12E-12 |
|  | 50S ribosomal protein L31 | EY04_RS01555 | COG0254 | J | -1.89 | 1.25E-03 |
|  | 30S ribosomal protein S2 | EY04_RS05150 | COG0052 | J | -1.89 | 2.40E-11 |
|  | 30S ribosomal protein S14 | EY04_RS28075 | COG0199 | J | -2.00 | 5.92E-10 |
|  | 50S ribosomal protein L2 | EY04_RS32900 | COG0090 | J | -2.08 | 8.17E-18 |
|  | 50S ribosomal protein L32 | EY04_RS32155 | COG0333 | J | -2.08 | 2.43E-22 |
|  | 50S ribosomal protein L14 | EY04_RS28090 | COG0093 | J | -2.13 | 2.74E-18 |
|  | 30S ribosomal protein S10 | EY04_RS28145 | COG0051 | J | -2.19 | 2.52E-11 |
|  | 50S ribosomal protein L5 | EY04_RS28080 | COG0094 | J | -2.21 | 2.23E-20 |
|  | 50S ribosomal protein L7/L12 | EY04_RS28180 | COG0222 | J | -2.23 | 1.83E-11 |
|  | 50S ribosomal protein L24 | EY04_RS28085 | COG0198 | J | -2.24 | 1.37E-19 |
|  | Elongation factor Tu | EY04_RS28215 | COG0050 | J | -2.25 | 1.19E-21 |
|  | Conjugal transfer protein TraR | EY04_RS11635 | COG1734 | J | -2.27 | 5.73E-05 |
|  | 50S ribosomal protein L9 | EY04_RS02220 | COG0359 | J | -2.30 | 5.64E-17 |
|  | 50S ribosomal protein L16 | EY04_RS28105 | COG0197 | J | -2.31 | 2.26E-25 |
|  | 30S ribosomal protein S12 | EY04_RS28165 | COG0048 | J | -2.34 | 2.48E-36 |
|  | 30S ribosomal protein S19 | EY04_RS28120 | COG0185 | J | -2.40 | 8.99E-23 |
|  | 30S ribosomal protein S17 | EY04_RS28095 | COG0186 | J | -2.43 | 5.59E-20 |
|  | 50S ribosomal protein L10 | EY04_RS28185 | COG0244 | J | -2.44 | 5.01E-22 |
|  | 30S ribosomal protein S7 | EY04_RS28160 | COG0049 | J | -2.46 | 2.22E-25 |
|  | 30S ribosomal protein S3 | EY04_RS28110 | COG0092 | J | -2.48 | 3.80E-39 |
|  | 16S rRNA processing protein RimM | EY04_RS04770 | COG0806 | J | -2.49 | 1.55E-30 |
|  | 50S ribosomal protein L22 | EY04_RS28115 | COG0091 | J | -2.50 | 2.79E-29 |
|  | 50S ribosomal protein L23 | EY04_RS28130 | COG0089 | J | -2.59 | 3.44E-21 |
|  | 30S ribosomal protein S6 | EY04_RS02205 | COG0360 | J | -2.64 | 6.30E-29 |
|  | 50S ribosomal protein L21 | EY04_RS26510 | COG0261 | J | -2.65 | 2.84E-21 |
|  | 50S ribosomal protein L3 | EY04_RS28140 | COG0087 | J | -2.67 | 9.75E-27 |
|  | 30S ribosomal protein S18 | EY04_RS02210 | COG0238 | J | -2.69 | 2.58E-17 |
|  | 50S ribosomal protein L21 | EY04_RS16195 | cl09109 | - | -2.71 | 8.05E-06 |
|  | 50S ribosomal protein L29 | EY04_RS28100 | COG0255 | J | -2.77 | 6.23E-27 |
|  | 50S ribosomal protein L28 | EY04_RS29730 | COG0227 | J | -2.86 | 2.07E-25 |
|  | 50S ribosomal protein L27 | EY04_RS26505 | COG0211 | J | -2.87 | 7.49E-16 |
|  | Elongation factor G | EY04_RS28155 | COG0480 | J | -2.88 | 9.32E-34 |
|  | Glutaminase | EY04_RS09740 | COG0252 | JU | -2.93 | 5.94E-22 |
|  | Elongation factor Tu | EY04_RS28150 | COG0050 | J | -3.07 | 7.71E-38 |
|  | 50S ribosomal protein L33 | EY04_RS29735 | COG0267 | J | -3.24 | 4.67E-35 |
|  | 50S ribosomal protein L4 | EY04_RS32905 | COG0088 | J | -3.34 | 1.00E-59 |
|  | Tryptophan synthase subunit alpha | EY04_RS11035 | cl27535 | - | -4.76 | 2.65E-111 |
| Transcription | LysR family transcriptional regulator | EY04_RS14120 | COG0583 | K | 2.70 | 2.98E-06 |
|  | Transcriptional regulator | EY04_RS14845 | - | - | 2.60 | 6.78E-06 |
|  | DNA-binding protein | EY04_RS13005 | COG1167 | KE | 2.49 | 2.79E-03 |
|  | Transcriptional regulator | EY04_RS29740 | COG3450 | K | 1.92 | 4.95E-05 |
|  | Histidine kinase | EY04_RS02720 | cl27674 | - | 1.91 | 2.06E-03 |
|  | AraC family transcriptional regulator | EY04_RS15465 | COG2207 | K | 1.91 | 1.58E-03 |
|  | Histidine kinase | EY04_RS26205 | cl01472 | - | 1.67 | 1.92E-03 |
|  | MarR family transcriptional regulator | EY04_RS07295 | COG1846 | K | -1.50 | 5.08E-06 |
|  | Cold-shock protein | EY04_RS23025 | COG1278 | K | -1.51 | 1.64E-06 |
|  | GntR family transcriptional regulator | EY04_RS13280 | COG1167 | KE | -1.53 | 1.64E-03 |
|  | Cro/Cl family transcriptional regulator | EY04_RS18125 | COG1396 | K | -1.68 | 4.09E-06 |
|  | LysR family transcriptional regulator | EY04_RS27730 | COG0583 | K | -1.69 | 1.05E-03 |
|  | Fis family transcriptional regulator | EY04_RS29535 | cl28069 | - | -1.87 | 1.06E-06 |
|  | AraC family transcriptional regulator | EY04_RS15230 | COG2207 | K | -2.07 | 6.33E-04 |
|  | RNA polymerase sigma factor RpoS | EY04_RS05305 | COG0568 | K | -2.13 | 3.60E-17 |
|  | Transcriptional regulator | EY04_RS20895 | - | - | -2.22 | 2.23E-03 |
|  | Hypothetical protein | EY04_RS12650 | COG1842 | KT | -2.28 | 3.12E-07 |
|  | Transcriptional regulator | EY04_RS22860 | COG1737 | K | -2.60 | 3.32E-15 |
|  | LuxR family transcriptional regulator CsaR | EY04_RS11855 | COG2771 | K | -2.64 | 4.88E-09 |
|  | Transcriptional regulator PhzR | EY04_RS25710 | COG2771 | R | -2.71 | 1.06E-30 |
|  | GntR family transcriptional regulator | EY04_RS23170 | COG2186 | K | -3.18 | 9.31E-23 |
|  | MarR family transcriptional regulator | EY04_RS19240 | COG1846 | K | -3.22 | 1.42E-03 |
|  | ArsR family transcriptional regulator | EY04_RS05450 | - | - | -3.88 | 4.77E-05 |
|  | PadR family transcriptional regulator | EY04_RS24465 | COG1695 | K | -4.12 | 1.22E-25 |
| Replication, recombination and repair | Dipicolinate synthase | EY04_RS04595 | COG0776 | L | -1.76 | 1.10E-14 |
|  | Hypothetical protein | EY04_RS29720 | COG2003 | L | -1.83 | 4.44E-08 |
|  | Nuclease | EY04_RS01560 | COG1525 | L | -1.84 | 1.36E-03 |
|  | RNA helicase | EY04_RS21020 | COG0513 | L | -2.13 | 3.20E-10 |
|  | Competence protein ComEA | EY04_RS21285 | COG1555 | L | -3.74 | 8.79E-10 |
| Cell wall/membrane/envelope biogenesis | N-acetylmuramoyl-L-alanine amidase | EY04_RS29170 | COG0860 | M | 6.09 | 1.02E-07 |
|  | Phospholipase | EY04_RS15425 | COG3511 | M | 2.90 | 7.37E-03 |
|  | Hypothetical protein | EY04_RS02370 | COG2982 | M | 1.75 | 5.26E-04 |
|  | ADP-heptose--LPS heptosyltransferase | EY04_RS01850 | COG0859 | M | 1.62 | 8.17E-03 |
|  | Channel protein TolC | EY04_RS18285 | COG1538 | M | 1.62 | 8.23E-03 |
|  | Hypothetical protein | EY04_RS14305 | COG1520 | M | -1.67 | 5.26E-12 |
|  | RND transporter | EY04_RS00160 | COG1538 | M | -1.79 | 1.00E-04 |
|  | Porin | EY04_RS24490 | COG3659 | M | -2.07 | 1.17E-03 |
|  | Glucosamine--fructose-6-phosphate aminotransferase | EY04_RS28955 | COG0449 | M | -2.08 | 1.26E-06 |
|  | Porin | EY04_RS08860 | COG2885 | M | -2.10 | 6.58E-26 |
|  | Membrane protein insertase | EY04_RS28870 | COG0706 | M | -2.13 | 6.01E-17 |
|  | Multidrug RND transporter | EY04_RS19235 | COG1538 | M | -2.34 | 5.18E-10 |
|  | Acriflavin resistance protein AcrA | EY04_RS17230 | COG0845 | MV | -2.59 | 1.85E-11 |
|  | N-acetylmuramoyl-L-alanine amidase | EY04_RS05345 | COG3023 | M | -3.81 | 2.89E-28 |
| Cell motility | Chemotaxis protein | EY04_RS16315 | COG0840 | NT | 4.06 | 6.96E-03 |
|  | Chemotaxis protein | EY04_RS19605 | COG0840 | NT | 2.83 | 8.69E-03 |
|  | Chemotaxis protein | EY04_RS00055 | COG0840 | NT | 2.07 | 6.46E-05 |
|  | Chemotaxis protein CheW | EY04_RS20840 | COG0835 | NT | 1.96 | 4.67E-07 |
|  | Chemotaxis protein | EY04_RS11640 | COG0840 | NT | 1.92 | 4.89E-03 |
|  | Flagellin | EY04_RS07580 | COG1344 | N | 1.85 | 1.07E-38 |
|  | Chemotaxis protein | EY04_RS01440 | COG0840 | NT | 1.85 | 4.02E-03 |
|  | Chemotaxis protein | EY04_RS22955 | COG0840 | NT | 1.82 | 6.91E-03 |
|  | Chemotaxis protein | EY04_RS09145 | COG0840 | NT | 1.61 | 5.33E-06 |
|  | Flagellar motor protein MotD | EY04_RS07755 | COG1360 | N | 1.60 | 2.85E-03 |
|  | Membrane protein MotY | EY04_RS24205 | COG2885 | M | 2.34 | 9.76E-04 |
|  | Chemotaxis protein | EY04_RS22505 | COG0840 | NT | 1.54 | 3.78E-03 |
|  | Flagellar basal body rod protein FlgB | EY04_RS22220 | COG1815 | N | -1.56 | 9.24E-09 |
|  | Flagellar basal body rod protein FlgG | EY04_RS07465 | COG4786 | N | -1.68 | 3.63E-15 |
|  | Flagellar hook protein FlgE | EY04_RS22205 | COG1749 | N | -2.00 | 8.13E-26 |
| Posttranslational modification, protein turnover, chaperones | Protease | EY04_RS29100 | COG0330 | O | 3.38 | 1.25E-05 |
|  | Membrane protein | EY04_RS29095 | COG0330 | O | 2.70 | 1.38E-09 |
|  | Membrane protein | EY04_RS29105 | COG0330 | O | 2.36 | 9.99E-05 |
|  | Peptidylprolyl isomerase | EY04_RS19470 | COG0760 | O | 2.29 | 5.31E-08 |
|  | Molecular chaperone HscC | EY04_RS09520 | COG0443 | O | -1.57 | 4.35E-03 |
|  | Chaperone protein HscA | EY04_RS24700 | COG0443 | O | -1.59 | 1.03E-04 |
|  | Co-chaperone HscB | EY04_RS24705 | COG1076 | O | -1.60 | 6.58E-05 |
|  | Thiol:disulfide interchange protein | EY04_RS18615 | COG0526 | O | -1.67 | 8.13E-03 |
|  | Molecular chaperone GroEL | EY04_RS24125 | COG0459 | O | -2.02 | 2.72E-12 |
|  | Thioredoxin reductase | EY04_RS04070 | COG0492 | O | -2.07 | 2.53E-07 |
|  | Trigger factor | EY04_RS19500 | COG0544 | O | -2.14 | 1.18E-20 |
|  | ATPase AAA | EY04_RS29530 | COG0542 | O | -2.31 | 1.49E-16 |
|  | Hypothetical protein | EY04_RS14340 | COG2234 | O | -2.83 | 7.44E-13 |
| Inorganic ion transport and metabolism | Metal ABC transporter substrate-binding protein | EY04_RS29150 | COG0803 | P | 7.82 | 1.73E-09 |
|  | Zinc ABC transporter permease | EY04_RS29155 | COG1108 | P | 6.52 | 2.27E-06 |
|  | TonB-dependent receptor | EY04_RS24485 | COG1629 | P | 5.80 | 5.78E-16 |
|  | AcsD protein | EY04_RS15400 | cl27184 | - | 5.07 | 3.04E-07 |
|  | Iron ABC transporter substrate-binding protein | EY04_RS02495 | COG0614 | P | 4.82 | 6.55E-04 |
|  | TonB-dependent receptor | EY04_RS02505 | COG1629 | P | 4.77 | 2.52E-06 |
|  | Histidinol phosphatase | EY04_RS02500 | COG1120 | PH | 4.49 | 2.01E-03 |
|  | Achromobactin-binding protein | EY04_RS15370 | COG0614 | P | 4.41 | 2.77E-03 |
|  | M,olybdenum ABC transporter permease | EY04_RS16615 | COG4149 | P | 4.10 | 7.07E-03 |
|  | TonB-dependent receptor | EY04_RS15410 | COG1629 | P | 4.07 | 9.82E-26 |
|  | ABC transporter permease | EY04_RS02490 | COG0609 | P | 4.04 | 7.04E-03 |
|  | AcsA protein | EY04_RS15375 | cl27184 | - | 3.76 | 9.24E-07 |
|  | admium ABC transporter ATPase | EY04_RS29110 | COG2217 | P | 3.36 | 7.15E-11 |
|  | Molybdenum ABC transporter ATP-binding protein | EY04_RS16620 | COG4148 | P | 2.61 | 4.66E-05 |
|  | AcsC protein | EY04_RS15385 | cl27184 | - | 2.61 | 5.29E-04 |
|  | Membrane protein | EY04_RS28490 | COG1108 | P | 2.22 | 1.80E-03 |
|  | Cystathionine gamma-synthase | EY04_RS30070 | COG0672 | P | 2.10 | 7.08E-09 |
|  | Peptide ABC transporter substrate-binding protein | EY04_RS00045 | COG3712 | PT | 2.09 | 4.41E-05 |
|  | Pyoverdine biosynthesis protein | EY04_RS20780 | - | - | 2.06 | 2.21E-20 |
|  | Ee2+ Zn2+ uptake regulation protein | EY04_RS13445 | cl21459 | - | 1.82 | 8.81E-14 |
|  | Iron dicitrate transport regulator FecR | EY04_RS17435 | COG3712 | PT | 1.52 | 1.84E-06 |
|  | ABC transporter substrate-binding protein | EY04_RS21760 | COG1840 | P | -1.63 | 1.90E-06 |
|  | Nuclease PIN | EY04_RS05935 | COG0306 | P | -1.77 | 3.06E-04 |
|  | Catalase | EY04_RS28010 | COG0753 | P | -1.82 | 3.58E-07 |
|  | Metal ABC transporter ATPase | EY04_RS02825 | COG2217 | P | -2.11 | 3.33E-05 |
|  | FAD-binding protein | EY04_RS24470 | COG2375 | P | -2.28 | 2.05E-19 |
|  | Carbonate dehydratase | EY04_RS25965 | COG0288 | P | -2.48 | 2.84E-12 |
|  | Catalase | EY04_RS26655 | COG0753 | P | -2.90 | 3.65E-05 |
|  | Sulfite reductase | EY04_RS13220 | COG0155 | P | -3.31 | 1.27E-58 |
| Secondary metabolites biosynthesis, transport and catabolism | Peptide synthase | EY04_RS20770 | COG1020 | Q | 2.39 | 1.19E-39 |
|  | Acyl-homoserine lactone acylase subunit beta | EY04_RS13450 | COG2366 | Q | 1.94 | 4.53E-09 |
|  | Peptide synthase | EY04_RS20315 | COG1020 | Q | 1.66 | 3.45E-12 |
|  | Peptide synthase | EY04_RS20305 | COG1020 | Q | 1.51 | 1.90E-12 |
|  | Homogentisate 1,2-dioxygenase | EY04_RS04180 | COG3508 | Q | -1.68 | 1.11E-04 |
|  | 2Fe-2S ferredoxin PrnD | EY04_RS17635 | cl28556 | - | -1.86 | 1.03E-09 |
|  | Tryptophan halogenase PrnA | EY04_RS17650 | cl26176 | - | -1.93 | 7.18E-19 |
|  | 2,5-diketo-D-gluconic acid reductase | EY04_RS20220 | COG0656 | Q | -2.33 | 3.45E-03 |
|  | 3-oxoacyl-ACP synthase | EY04_RS20365 | cl28397 | - | -2.51 | 1.91E-07 |
|  | FAD-dependent oxidoreductase PrnC | EY04_RS17640 | cl27554 | - | -2.63 | 3.31E-32 |
|  | Ring-cleavage extradiol dioxygenase | EY04_RS14300 | COG0346 | Q | -3.29 | 3.39E-24 |
|  | (2Fe-2S)-binding protein HcnA | EY04_RS11540 | cl09928 | - | -3.42 | 2.72E-27 |
|  | Serine 3-dehydrogenase | EY04_RS15795 | COG2931 | Q | -3.57 | 3.67E-68 |
|  | Serine 3-dehydrogenase | EY04_RS11085 | COG2931 | Q | -4.24 | 6.04E-123 |
|  | Phenazine biosynthesis protein PhzB | EY04_RS25720 | - | - | -6.21 | 1.45E-94 |
|  | Phenazine biosynthesis protein PhzG | EY04_RS25745 | cl25685 | - | -6.21 | 2.25E-105 |
|  | Anthranilate synthase PhzE | EY04_RS25735 | cl27696 | - | -6.27 | 1.55E-154 |
|  | Phospho-2-dehydro-3-deoxyheptonate aldolase PhzC | EY04_RS25725 | cl03230 | - | -6.59 | 1.24E-150 |
|  | Phenazine biosynthesis protein PhzA | EY04_RS25715 | cl09109 | - | -6.68 | 1.69E-122 |
|  | Phenazine biosynthesis protein PhzO | EY04_RS25750 | cl26441 | - | -7.27 | 0.00E+00 |
|  | Isochorismatase | EY04_RS25730 | COG1535 | Q | -7.61 | 4.78E-63 |
|  | 2,3-dihydro-3-hydroxyanthranilate isomerase | EY04_RS25740 | COG0384 | R | -6.28 | 3.10E-121 |
| General function prediction only | Hypothetical protein | EY04_RS29185 | COG0523 | R | 8.07 | 4.76E-10 |
|  | Carbonate dehydratase | EY04_RS29140 | COG0663 | R | 7.63 | 5.24E-09 |
|  | Cobalt transporter | EY04_RS18445 | COG5446 | R | 2.72 | 5.62E-04 |
|  | Carbon-nitrogen hydrolase | EY04_RS03865 | COG0388 | R | 1.90 | 1.10E-03 |
|  | Phosphoesterase | EY04_RS07385 | COG0613 | R | 1.75 | 1.51E-04 |
|  | RND transporter | EY04_RS12855 | COG1033 | R | 1.57 | 6.29E-03 |
|  | Cobalamin biosynthesis protein CobW | EY04_RS18435 | COG0523 | R | 1.53 | 7.31E-03 |
|  | Isomerase | EY04_RS20135 | COG0384 | R | -1.83 | 9.23E-04 |
|  | Hypothetical protein | EY04_RS30945 | COG2353 | R | -1.85 | 3.67E-15 |
|  | Fe(II)-dependent oxygenase | EY04_RS03630 | COG3128 | R | -2.01 | 2.31E-10 |
|  | Cytochrome D ubiquinol oxidase subunit II | EY04_RS05545 | COG1611 | R | -2.20 | 1.15E-12 |
|  | Glutamine amidotransferase | EY04_RS06140 | COG0693 | R | -2.83 | 4.20E-18 |
|  | Arylsulfate sulfotransferase | EY04_RS06145 | COG4321 | R | -3.26 | 5.87E-13 |
| Function unknown | GTP cyclohydrolase | EY04_RS29165 | cl00642 | - | 7.73 | 6.92E-09 |
|  | Hypothetical protein | EY04_RS29190 | - | - | 7.52 | 6.40E-09 |
|  | Nickel uptake transporter family protein | EY04_RS15510 | - | - | 6.48 | 3.32E-06 |
|  | Manganese ABC transporter ATP-binding protein | EY04_RS29160 | cl28181 | - | 6.26 | 4.14E-06 |
|  | Membrane protein | EY04_RS15505 | cl21495 | - | 5.83 | 7.59E-07 |
|  | Cobalamin biosynthesis protein CobW | EY04_RS29195 | cl26870 | - | 5.43 | 6.87E-05 |
|  | Porin | EY04_RS15500 | - | - | 5.31 | 2.05E-04 |
|  | Hypothetical protein | EY04_RS18130 | - | - | 5.00 | 4.20E-04 |
|  | Glutamine synthetase | EY04_RS29175 | - | - | 4.94 | 2.63E-05 |
|  | Hypothetical protein | EY04_RS28530 | - | - | 4.70 | 1.63E-03 |
|  | Peptidase S8 and S53 subtilisin kexin sedolisin | EY04_RS20470 | - | - | 4.38 | 3.06E-03 |
|  | MFS transporter | EY04_RS29750 | - | - | 4.33 | 2.94E-03 |
|  | Cobalamin biosynthesis protein CobE | EY04_RS18450 | - | - | 4.25 | 4.30E-03 |
|  | Hypothetical protein | EY04_RS23360 | - | - | 4.12 | 6.19E-03 |
|  | Hypothetical protein | EY04_RS13355 | - | - | 3.67 | 4.44E-03 |
|  | (Fe-S)-binding protein | EY04_RS09565 | cl25381 | - | 3.52 | 6.67E-05 |
|  | Hypothetical protein | EY04_RS27905 | - | - | 3.15 | 5.91E-03 |
|  | Hypothetical protein | EY04_RS23335 | - | - | 3.09 | 8.83E-16 |
|  | Hypothetical protein | EY04_RS25435 | - | - | 3.00 | 1.37E-04 |
|  | Hemolysin D | EY04_RS18300 | cl25633 | - | 2.96 | 4.08E-26 |
|  | Hypothetical protein | EY04_RS11660 | - | - | 2.92 | 6.51E-03 |
|  | Hypothetical protein | EY04_RS05895 | - | - | 2.81 | 8.69E-08 |
|  | Hypothetical protein | EY04_RS06970 | - | - | 2.59 | 2.56E-07 |
|  | Hypothetical protein | EY04_RS23340 | - | - | 2.56 | 9.53E-34 |
|  | Membrane protein | EY04_RS07335 | cl01879 | - | 2.43 | 1.55E-03 |
|  | Hypothetical protein | EY04_RS11645 | - | - | 2.42 | 1.77E-03 |
|  | Hypothetical protein | EY04_RS15455 | - | - | 2.29 | 5.29E-08 |
|  | Hypothetical protein | EY04_RS29765 | cl23975 | - | 2.26 | 4.42E-03 |
|  | Hypothetical protein | EY04_RS27900 | - | - | 2.26 | 8.13E-04 |
|  | Hypothetical protein | EY04_RS20845 | - | - | 2.25 | 5.49E-04 |
|  | Pilus assembly protein PilZ | EY04_RS23905 | - | - | 2.24 | 8.85E-03 |
|  | PasA protein | EY04_RS09230 | - | - | 2.23 | 2.63E-06 |
|  | Methyltransferase | EY04_RS18230 | cl28093 | - | 2.16 | 1.32E-04 |
|  | CAAX protease | EY04_RS09150 | - | - | 2.11 | 6.10E-03 |
|  | Filamentous hemagglutinin | EY04_RS20950 | - | - | 2.03 | 8.39E-08 |
|  | Hypothetical protein | EY04_RS06510 | - | - | 2.00 | 6.29E-03 |
|  | Membrane protein | EY04_RS06125 | cl01535 | - | 1.93 | 3.07E-03 |
|  | Nitrate reductase | EY04_RS01990 | cl00456 | - | 1.84 | 1.90E-03 |
|  | Multifunctional fatty acid oxidation complex subunit alpha | EY04_RS09180 | - | - | 1.76 | 2.26E-03 |
|  | Hypothetical protein | EY04_RS14850 | - | - | 1.74 | 3.60E-03 |
|  | Sterol desaturase | EY04_RS18625 | - | - | 1.73 | 7.49E-03 |
|  | Hypothetical protein | EY04_RS14855 | - | - | 1.69 | 1.16E-03 |
|  | Membrane protein | EY04_RS22140 | - | - | 1.67 | 3.02E-05 |
|  | Hypothetical protein | EY04_RS08660 | - | - | 1.65 | 1.69E-04 |
|  | Hypothetical protein | EY04_RS12830 | cl01215 | - | 1.64 | 8.39E-03 |
|  | Membrane protein | EY04_RS02715 | cl26578 | - | 1.58 | 3.53E-03 |
|  | Pilus assembly protein | EY04_RS23310 | cl28318 | - | 1.57 | 4.23E-04 |
|  | Cyclic peptide transporter | EY04_RS20295 | cl26602 | - | 1.55 | 2.40E-06 |
|  | Alpha/beta hydrolase | EY04_RS24355 | cl21494 | - | -1.50 | 2.40E-05 |
|  | ATPase | EY04_RS30435 | - | - | -1.54 | 3.58E-05 |
|  | Hypothetical protein | EY04_RS08075 | - | - | -1.54 | 2.24E-05 |
|  | Hypothetical protein | EY04_RS03330 | cl22854 | - | -1.54 | 6.41E-13 |
|  | Membrane protein | EY04_RS02215 | - | - | -1.54 | 5.57E-05 |
|  | Membrane protein | EY04_RS09710 | cl01427 | - | -1.55 | 3.32E-04 |
|  | Serine protease | EY04_RS15765 | cl22877 | - | -1.55 | 2.83E-06 |
|  | Hypothetical protein | EY04_30695 | - | - | -1.56 | 2.32E-08 |
|  | Hypothetical protein | EY04_30690 | - | - | -1.57 | 1.49E-05 |
|  | Acetyltransferase | EY04_RS00190 | cl26092 | - | -1.57 | 2.77E-04 |
|  | Hypothetical protein | EY04_RS18385 | cl25370 | - | -1.60 | 6.33E-08 |
|  | Hypothetical protein | EY04_RS05495 | - | - | -1.62 | 7.82E-03 |
|  | Hypothetical protein | EY04_RS14315 | - | - | -1.63 | 3.05E-06 |
|  | Hypothetical protein | EY04_RS11235 | cl19824 | - | -1.64 | 5.67E-03 |
|  | Hypothetical protein | EY04_RS25255 | - | - | -1.65 | 1.73E-04 |
|  | Membrane protein | EY04_RS22175 | - | - | -1.67 | 1.26E-05 |
|  | Serine protease | EY04_RS19790 | cl27557 | - | -1.69 | 5.59E-11 |
|  | Membrane protein | EY04_RS20045 | - | - | -1.72 | 1.59E-07 |
|  | Hypothetical protein | EY04_RS13205 | - | - | -1.73 | 1.46E-05 |
|  | Hypothetical protein | EY04_RS09805 | - | - | -1.76 | 4.44E-03 |
|  | Hypothetical protein | EY04_RS05515 | - | - | -1.76 | 1.78E-03 |
|  | Hypothetical protein | EY04_RS20450 | cl01604 | - | -1.77 | 1.69E-07 |
|  | Ion channel protein Tsx | EY04_RS10235 | cl04114 | - | -1.78 | 7.41E-03 |
|  | Hypothetical protein | EY04_RS12375 | - | - | -1.90 | 1.64E-08 |
|  | CrfX protein | EY04_RS08845 | - | - | -1.90 | 8.05E-06 |
|  | ZapA | EY04_RS01800 | cl01146 | - | -1.90 | 6.91E-03 |
|  | Hypothetical protein | EY04_RS14665 | - | - | -1.92 | 3.63E-07 |
|  | Hypothetical protein | EY04_RS10670 | - | - | -1.94 | 2.95E-03 |
|  | Hypothetical protein | EY04_RS10230 | cl00303 | - | -1.95 | 2.46E-04 |
|  | Hypothetical protein | EY04_RS13695 | - | - | -1.97 | 6.50E-07 |
|  | Adenosylmethionine-8-amino-7-oxononanoate aminotransferase | EY04_RS24350 | cl28400 | - | -1.97 | 3.35E-06 |
|  | Metal-binding protein | EY04_RS08435 | cl00616 | - | -1.98 | 1.83E-15 |
|  | Hypothetical protein | EY04_RS30255 | - | - | -1.99 | 7.42E-04 |
|  | Molecular chaperone DnaJ | EY04_RS09515 | - | - | -2.00 | 8.99E-05 |
|  | Hypothetical protein | EY04_RS29845 | - | - | -2.04 | 7.51E-10 |
|  | Hypothetical protein | EY04_RS00195 | - | - | -2.07 | 7.69E-04 |
|  | Hypothetical protein | EY04_RS29515 | - | - | -2.09 | 1.29E-06 |
|  | Nucleotidyltransferase | EY04_RS15975 | - | - | -2.10 | 2.50E-09 |
|  | Hypothetical protein | EY04_RS31370 | - | - | -2.15 | 8.88E-03 |
|  | Hypothetical protein | EY04_RS03745 | cl01077 | - | -2.16 | 1.32E-08 |
|  | Hypothetical protein | EY04_RS20865 | - | - | -2.17 | 2.65E-06 |
|  | Hypothetical protein | EY04_RS12655 | cl01551 | - | -2.20 | 1.31E-07 |
|  | Hypothetical protein | EY04_RS08540 | - | - | -2.20 | 5.89E-07 |
|  | Nuclease | EY04_01095 | - | - | -2.21 | 2.79E-09 |
|  | Hypothetical protein | EY04_RS13210 | - | - | -2.24 | 8.39E-05 |
|  | Hypothetical protein | EY04_RS11560 | - | - | -2.25 | 7.08E-17 |
|  | Sucrase | EY04_RS24855 | - | - | -2.25 | 4.77E-05 |
|  | Hypothetical protein | EY04_RS17880 | - | - | -2.28 | 8.73E-03 |
|  | Hypothetical protein | EY04_RS25960 | - | - | -2.31 | 2.60E-31 |
|  | PsiE family protein | EY04_RS04240 | cl01264 | - | -2.32 | 2.12E-07 |
|  | Hypothetical protein | EY04_RS13890 | - | - | -2.32 | 8.13E-19 |
|  | Glucokinase | EY04_RS22910 | cl17037 | - | -2.35 | 9.42E-21 |
|  | Hypothetical protein | EY04_RS13340 | - | - | -2.36 | 9.32E-17 |
|  | Hypothetical protein | EY04_RS05455 | cl26558 | - | -2.38 | 8.71E-10 |
|  | 2-hydroxyacid dehydrogenase | EY04_RS05460 | cl19561 | - | -2.40 | 2.56E-07 |
|  | Hypothetical protein | EY04_RS05620 | - | - | -2.46 | 1.69E-05 |
|  | Peptidase M4 | EY04_RS22550 | cl26847 | - | -2.50 | 5.06E-03 |
|  | Transporter | EY04_RS30345 | cl01440 | - | -2.52 | 9.67E-05 |
|  | Phosphatidylserine decarboxylase | EY04_RS10850 | cl03656 | - | -2.52 | 2.08E-12 |
|  | Calcium transporter ChaC | EY04_RS30155 | cl26085 | - | -2.54 | 6.76E-15 |
|  | Hypothetical protein | EY04_RS28565 | - | - | -2.55 | 1.78E-15 |
|  | Hypothetical protein | EY04_03550 | - | - | -2.56 | 3.74E-44 |
|  | Hypothetical protein | EY04_RS12635 | - | - | -2.58 | 4.08E-05 |
|  | Hypothetical protein | EY04_RS32550 | - | - | -2.58 | 1.64E-03 |
|  | Phosphatidylcholine-hydrolyzing phospholipase | EY04_RS03775 | - | - | -2.60 | 2.14E-12 |
|  | Signal peptide protein | EY04_RS29545 | cl00062 | - | -2.63 | 1.12E-13 |
|  | Lipoprotein | EY04_RS13885 | - | - | -2.67 | 1.48E-27 |
|  | Ligand-gated channel protein | EY04_RS03625 | cl25796 | - | -2.68 | 1.15E-06 |
|  | Hypothetical protein | EY04_RS10060 | - | - | -2.69 | 1.06E-30 |
|  | Hypothetical protein | EY04_RS11535 | - | - | -2.71 | 2.42E-10 |
|  | Hypothetical protein | EY04_RS19325 | - | - | -2.73 | 3.76E-09 |
|  | Hypothetical protein | EY04_RS00390 | - | - | -2.73 | 3.03E-12 |
|  | Hypothetical protein | EY04_RS29510 | - | - | -2.74 | 2.73E-20 |
|  | Hypothetical protein | EY04_RS16685 | - | - | -2.79 | 4.73E-31 |
|  | Hypothetical protein | EY04_RS31325 | - | - | -2.80 | 2.21E-18 |
|  | Porin | EY04_RS23775 | - | - | -2.87 | 3.06E-62 |
|  | Hypothetical protein | EY04_RS14295 | - | - | -2.88 | 2.09E-33 |
|  | Type VI secretion protein | EY04_RS29540 | - | - | -2.89 | 6.67E-05 |
|  | Hypothetical protein | EY04_RS18225 | - | - | -2.97 | 5.89E-04 |
|  | Hypothetical protein | EY04_RS29505 | - | - | -3.02 | 7.01E-15 |
|  | Hypothetical protein | EY04_RS09900 | - | - | -3.03 | 3.74E-07 |
|  | Mercuric reductase | EY04_07515 | - | - | -3.06 | 1.47E-09 |
|  | Hypothetical protein | EY04_RS05465 | cl15796 | - | -3.20 | 9.36E-08 |
|  | Hypothetical protein | EY04_RS12155 | - | - | -3.20 | 4.78E-29 |
|  | Hypothetical protein | EY04_RS13225 | cl01526 | - | -3.24 | 1.82E-28 |
|  | Hypothetical protein | EY04_RS05430 | - | - | -3.26 | 3.18E-04 |
|  | Type VI secretion protein | EY04_RS29490 | cl01402 | - | -3.27 | 2.20E-41 |
|  | R body protein RebB-like protein | EY04_RS00400 | - | - | -3.49 | 8.32E-36 |
|  | Hypothetical protein | EY04_RS05500 | - | - | -3.51 | 7.37E-07 |
|  | R body protein RebB-like protein | EY04_RS00405 | - | - | -3.83 | 9.43E-27 |
|  | Phospho-2-dehydro-3-deoxyheptonate aldolase | EY04_RS18145 | cl17225 | - | -3.88 | 1.81E-22 |
|  | Sugar isomerase | EY04_RS22895 | cl23840 | - | -3.89 | 1.98E-10 |
|  | Amidohydrolase | EY04_RS20460 | cl26822 | - | -3.98 | 2.69E-35 |
|  | dialkylrecorsinol condensing enzyme | EY04_RS20370 | - | - | -4.07 | 6.53E-06 |
|  | Leucyl aminopeptidase (aminopeptidase T) | EY04_RS07410 | - | - | -4.14 | 4.45E-46 |
|  | Hypothetical protein | EY04_RS05400 | - | - | -4.27 | 2.67E-12 |
|  | Hypothetical protein | EY04_32005 | - | - | -4.28 | 9.07E-18 |
|  | Methyltransferase | EY04_RS14345 | cl28097 | - | -4.39 | 5.47E-27 |
|  | Hypothetical protein | EY04_03130 | - | - | -4.40 | 3.66E-76 |
|  | Hypothetical protein | EY04_RS05505 | - | - | -4.52 | 1.17E-07 |
|  | Hypothetical protein | EY04_RS17125 | - | - | -4.62 | 1.15E-79 |
|  | Hypothetical protein | EY04_RS05350 | - | - | -4.68 | 6.30E-11 |
|  | Hypothetical protein | EY04_RS05385 | - | - | -4.90 | 1.26E-06 |
|  | Stress-induced protein YgiW | EY04_RS01630 | cl09930 | - | -4.91 | 2.77E-04 |
|  | Hypothetical protein | EY04_RS14645 | - | - | -5.27 | 2.64E-131 |
|  | Dockerin | EY04_RS05335 | - | - | -5.35 | 1.14E-71 |
|  | Hypothetical protein | EY04_RS24450 | - | - | -5.89 | 5.33E-77 |
|  | Hypothetical protein | EY04_RS23175 | - | - | -6.15 | 4.08E-72 |
|  | Hypothetical protein | EY04_RS23180 | - | - | -6.24 | 7.88E-65 |
|  | Hypothetical protein | EY04_RS24445 | - | - | -6.25 | 7.64E-77 |
|  | C4-dicarboxylate ABC transporter | EY04_RS21880 | cl04176 | - | -6.71 | 1.22E-91 |
|  | Hypothetical protein | EY04_RS05435 | - | - | -7.62 | 3.58E-08 |
|  | Hypothetical protein | EY04_RS23185 | - | - | -7.69 | 1.24E-129 |
|  | Hypothetical protein | EY04_RS23190 | - | - | -8.16 | 0.00E+00 |
| Signal transduction mechanisms | Diguanylate phosphodiesterase | EY04_RS15460 | COG2200 | T | 2.97 | 6.40E-31 |
|  | Chemotaxis protein CheY | EY04_RS22270 | COG0784 | T | 2.28 | 2.44E-08 |
|  | Diguanylate phosphodiesterase | EY04_RS05905 | COG2200 | T | 2.27 | 5.57E-07 |
|  | Chemotaxis protein | EY04_RS25870 | COG2202 | T | 2.13 | 2.26E-03 |
|  | Histidine kinase | EY04_RS10080 | COG2199 | T | 1.91 | 4.14E-03 |
|  | Iron dicitrate transport regulator FecR | EY04_RS10680 | cl27113 | - | 1.88 | 6.68E-09 |
|  | Chemotaxis protein CheY | EY04_RS29425 | COG0784 | T | 1.83 | 1.24E-05 |
|  | DeoR faimly transcriptional regulator | EY04_RS24510 | COG2200 | T | 1.71 | 4.58E-09 |
|  | Transcriptional regulator | EY04_RS28835 | COG0745 | TK | 1.61 | 3.74E-04 |
|  | Histidine kinase | EY04_RS02065 | - | - | 1.59 | 2.74E-04 |
|  | Histidine kinase | EY04_RS18280 | COG0642 | T | 1.56 | 1.03E-04 |
|  | Chemotaxis protein CheY | EY04_RS22905 | COG0745 | TK | -1.71 | 8.05E-05 |
|  | Histidine kinase | EY04_RS22900 | COG0642 | T | -1.78 | 2.18E-05 |
|  | Hypothetical protein | EY04_RS29585 | COG0790 | T | -2.30 | 1.68E-08 |
|  | GTP-binding protein TypA | EY04_RS01290 | COG1217 | T | -2.44 | 7.76E-34 |
|  | Serine/threonine protein kinase | EY04_RS29575 | COG0515 | T | -2.78 | 7.14E-06 |
|  | Acyl-homoserine-lactone synthase PhzI | EY04_RS25705 | cl17182 | - | -5.15 | 1.78E-152 |
|  | Acyl-homoserine-lactone synthase CsaI | EY04_RS11850 | cl17182 | - | -5.74 | 4.46E-38 |
| Intracellular trafficking, secretion, and vesicular transport | ShlB family hemolysin secretion/activation protein | EY04_RS20945 | COG2831 | U | 2.34 | 6.22E-03 |
|  | ATPase | EY04_RS02750 | COG4962 | UW | 2.22 | 3.49E-03 |
|  | Type VI secretion protein | EY04_RS29555 | COG3522 | U | -1.58 | 1.75E-04 |
|  | Type VI secretion protein | EY04_RS29520 | COG3519 | U | -1.69 | 1.57E-04 |
|  | Type VI secretion protein | EY04_RS29525 | COG3520 | U | -1.78 | 1.95E-03 |
|  | Hypothetical protein | EY04_RS01085 | COG4104 | U | -1.88 | 6.62E-03 |
|  | EvpB family type VI secretion protein | EY04_RS17870 | COG3517 | U | -2.07 | 1.30E-04 |
|  | Membrane protein | EY04_RS29560 | COG3455 | U | -2.36 | 1.15E-09 |
|  | Type VI secretion protein VasK | EY04_RS29565 | COG3523 | U | -2.72 | 2.45E-15 |
|  | Type VI secretion protein | EY04_RS29550 | COG3521 | U | -2.75 | 4.14E-06 |
|  | Type IV secretion protein Rhs | EY04_RS29580 | COG3501 | UXR | -2.93 | 1.25E-17 |
|  | Hypothetical protein | EY04_RS29500 | COG3518 | U | -3.20 | 4.47E-17 |
|  | Type IV secretion protein Rhs | EY04_RS00555 | COG3501 | UXR | -3.35 | 1.11E-12 |
|  | Type VI secretion protein | EY04_RS29495 | COG3517 | U | -3.64 | 7.66E-58 |
|  | Hypothetical protein | EY04_RS00560 | COG3157 | U | -6.13 | 8.56E-96 |
| Defense mechanisms | Peptide ABC transporter permease | EY04_RS27915 | COG0577 | V | 3.00 | 4.27E-04 |
|  | Peptidase C39 | EY04_RS18295 | COG2274 | V | 2.60 | 4.47E-26 |
|  | Peptidase C39 | EY04_RS18305 | COG2274 | V | 2.23 | 8.45E-16 |
|  | Secretion protein HylD | EY04_RS22815 | COG1566 | V | -1.58 | 6.88E-04 |
|  | Alkyl hydroperoxide reductase | EY04_RS16520 | COG0450 | V | -1.83 | 4.90E-06 |
|  | Hypothetical protein | EY04_RS05675 | COG2849 | V | -1.86 | 6.69E-04 |
|  | Beta-lactamase/D-alanine carboxypeptidase | EY04_RS20150 | COG1680 | V | -1.87 | 1.24E-03 |
|  | Membrane protein | EY04_RS00285 | COG1566 | V | -1.98 | 2.72E-03 |
|  | Transporter | EY04_RS17225 | COG0841 | V | -2.23 | 1.83E-12 |
|  | Hemolysin D | EY04_RS19230 | COG1566 | V | -2.86 | 2.36E-12 |
|  | DSBA oxidoreductase | EY04_RS00155 | COG1566 | V | -3.00 | 8.75E-06 |
|  | Pyocin R2, holin | EY04_RS05340 | - | - | -3.72 | 4.29E-14 |
| Mobilome: prophages, transposons | Tail protein | EY04_RS00200 | cl26890 | - | -2.30 | 6.50E-07 |
|  | Transposase | EY04_RS21920 | COG3464 | X | -2.39 | 2.50E-04 |
|  | Baseplate J protein | EY04_RS05480 | COG3299 | X | -2.42 | 2.27E-03 |
|  | Phage tail protein | EY04_RS05380 | - | - | -2.45 | 3.91E-06 |
|  | Phage tail protein | EY04_RS05395 | cl01390 | - | -2.54 | 4.11E-09 |
|  | Hypothetical protein | EY04_RS05425 | COG3500 | X | -2.60 | 1.48E-04 |
|  | Tail protein | EY04_RS05375 | cl26341 | - | -2.96 | 3.26E-23 |
|  | Tail protein | EY04_RS05420 | cl02088 | - | -3.02 | 3.41E-03 |
|  | Tail protein | EY04_RS05410 | - | - | -3.26 | 5.28E-19 |
|  | Baseplate J protein | EY04_RS05365 | cl01294 | - | -3.31 | 2.78E-14 |
|  | Tail protein | EY04_RS05370 | cl01817 | - | -3.34 | 3.04E-06 |
|  | Caudovirales tail fiber assembly protein | EY04_RS05330 | - | - | -4.02 | 2.95E-55 |
|  | Phage assembly protein | EY04_RS05470 | cl17812 | - | -4.16 | 3.83E-03 |
|  | Baseplate assembly protein | EY04_RS05355 | cl17812 | - | -4.36 | 3.38E-15 |
|  | Phage tail protein | EY04_RS05445 | - | - | -4.40 | 1.73E-10 |
|  | Tail protein | EY04_RS05390 | COG3497 | X | -4.49 | 2.05E-28 |
|  | Tail sheath protein | EY04_RS05440 | cl26913 | - | -4.51 | 8.89E-47 |
|  | sRNA | *rsmZ* |  |  | -3.57 | 7.59E-12 |
