# Supplementary material for: Investigation of the quorum-sensing regulon of the biocontrol bacterium Pseudomonas chlororaphis strain PA23
Source: PLoS One. 2020 Feb 28;15(2):e0226232. doi: 10.1371/journal.pone.0226232 (PMC7048289; doi:10.1371/journal.pone.0226232)
Supplement: S3 Table — (DOCX) [file pone.0226232.s006.docx]

**Table S3.** Differentially expressed genes in PA23-6863 relative to PA23 wild type

| Functional category | Predicted function | Locus tag | COG accession | COG category | Log2 fold change | Adjusted P-value |
| --- | --- | --- | --- | --- | --- | --- |
| Energy production and conversion | Branched-chain alpha-keto acid dehydrogenase subunit E2 | EY04_RS11260 | COG0508 | C | 3.83 | 1.15E-06 |
|  | Dihydrolipoamide dehydrogenase | EY04_RS11255 | COG1249 | C | 3.02 | 1.38E-05 |
|  | Membrane protein | EY04_RS15295 | COG0277 | C | 2.91 | 1.16E-18 |
|  | 2-oxoisovalerate dehydrogenase | EY04_RS11265 | COG0022 | C | 2.83 | 2.88E-04 |
|  | ATP synthase F0F1 subunit delta | EY04_RS28920 | COG0712 | C | -1.50 | 3.48E-06 |
|  | Cytochrome D ubiquinol oxidase subunit III | EY04_RS25240 | cl11473 | - | -1.58 | 2.65E-03 |
|  | ATP synthase F0F1 subunit epsilon | EY04_RS28940 | COG0355 | C | -1.65 | 2.74E-04 |
|  | Cytochrome oxidase subunit I | EY04_RS28555 | COG0843 | C | -1.71 | 7.71E-05 |
|  | Cytochrome D ubiquinol oxidase subunit I | EY04_RS26680 | COG1271 | C | -1.86 | 8.97E-04 |
|  | 2Fe-2S ferredoxin | EY04_RS24695 | COG0633 | C | -1.91 | 1.34E-03 |
|  | Glycerol kinase | EY04_RS24270 | COG0554 | C | -1.97 | 3.11E-05 |
|  | ATP synthase F0F1 subunit beta | EY04_RS28935 | COG0055 | C | -2.05 | 1.43E-10 |
|  | Glycerol-3-phosphate dehydrogenase | EY04_RS24280 | COG0578 | C | -2.27 | 1.49E-08 |
|  | 2-hydroxyacid dehydrogenase | EY04_RS22095 | COG1052 | CHR | -2.42 | 7.65E-03 |
|  | MFS transporter | EY04_RS16325 | COG1853 | C | -2.43 | 7.84E-05 |
|  | Flavin reductase | EY04_RS17620 | COG1853 | C | -3.36 | 2.07E-05 |
|  | (Fe-S)-binding protein | EY04_RS13285 | COG0348 | C | -4.56 | 2.86E-09 |
|  | cbb3-type cytochrome c oxidase subunit I | EY04_RS13215 | COG3278 | C | -4.81 | 1.37E-30 |
| Cell cycle control, cell division, chromosome partitioning | Cell division protein FtsA | EY04_RS25080 | COG0849 | D | 1.72 | 8.64E-06 |
| Amino acid transport and metabolism | Phosphoribosyl-AMP cyclohydrolase | EY04_RS29145 | COG0139 | E | 5.91 | 3.17E-05 |
|  | Diaminobutyrate--2-oxoglutarate aminotransferase | EY04_RS15405 | COG0160 | E | 5.45 | 1.66E-25 |
|  | Dihydrodipicolinate reductase | EY04_RS03440 | COG0289 | E | 3.17 | 9.71E-05 |
|  | Serine acetyltransferase | EY04_RS00710 | COG1045 | E | 3.06 | 4.75E-03 |
|  | FAD-dependent oxidoreductase | EY04_RS15450 | COG0665 | E | 2.72 | 8.12E-06 |
|  | Amino acid ABC transporter substrate-binding protein | EY04_RS04525 | COG0834 | ET | 2.60 | 6.39E-07 |
|  | Gamma-aminobutyrate transporter | EY04_RS01120 | COG1113 | E | 2.11 | 3.30E-05 |
|  | Cysteine synthase | EY04_RS22075 | COG0031 | E | 1.96 | 2.49E-03 |
|  | Aromatic amino acid aminotransferase | EY04_RS09450 | COG1448 | E | 1.95 | 2.48E-03 |
|  | Amino acid dehydrogenase | EY04_RS29785 | COG0665 | E | 1.93 | 6.44E-06 |
|  | Aspartate ammonia-lyase | EY04_RS29355 | COG1027 | E | 1.91 | 4.60E-03 |
|  | Isopropylmalate isomerase | EY04_RS09535 | COG0065 | E | 1.83 | 8.83E-06 |
|  | Glutamate synthase | EY04_RS18240 | COG0069 | E | 1.78 | 4.44E-03 |
|  | 4-aminobutyrate aminotransferase | EY04_RS00415 | COG0160 | E | 1.66 | 4.18E-04 |
|  | D-alanine/D-serine/glycine permease | EY04_RS24455 | COG1113 | E | 1.64 | 5.14E-03 |
|  | Branched-chain amino acid aminotransferase | EY04_RS11250 | COG0115 | EH | 1.62 | 1.06E-03 |
|  | Porin | EY04_RS10105 | COG0531 | E | -1.93 | 9.86E-03 |
|  | Indole-3-glycerol-phosphate synthase | EY04_RS31770 | COG0134 | E | -1.93 | 6.28E-04 |
|  | Spermidine/putrescine ABC transporter substrate-binding protein | EY04_RS31720 | COG0687 | E | -2.38 | 2.49E-03 |
|  | Diguanylate cyclase | EY04_RS23595 | COG0747 | E | -2.51 | 8.36E-08 |
|  | Lysine transporter LysE | EY04_RS08295 | COG1280 | E | -2.95 | 6.83E-03 |
|  | Carbamate kinase | EY04_RS22990 | COG0549 | E | -3.22 | 4.00E-06 |
|  | Amino acid APC transporter | EY04_RS22975 | COG0531 | E | -3.82 | 4.29E-12 |
|  | Lysine transporter LysE | EY04_RS07965 | COG1280 | E | -4.16 | 3.29E-03 |
|  | Ornithine carbamoyltransferase | EY04_RS22985 | COG0078 | E | -4.38 | 2.72E-17 |
|  | Hypothetical protein | EY04_RS07960 | COG1280 | E | -4.47 | 1.84E-05 |
|  | Arginine deiminase | EY04_RS22980 | COG2235 | E | -4.54 | 4.05E-21 |
|  | Lysine transporter LysE | EY04_RS07940 | COG1280 | E | -4.61 | 4.73E-05 |
|  | Hydrogen cyanide synthase HcnC | EY04_RS11550 | COG0665 | EQ | -5.65 | 3.82E-18 |
| Nucleotide transport and metabolism | Dihydroorotase | EY04_RS29135 | COG0044 | F | 6.65 | 1.21E-06 |
|  | Inorganic polyphosphate kinase | EY04_RS12815 | COG0061 | F | 1.65 | 6.24E-03 |
|  | Nucleoside diphosphate kinase | EY04_RS24685 | COG0105 | F | -2.46 | 1.19E-15 |
| Carbohydrate transport and metabolism | MFS transporter | EY04_RS15390 | COG2814 | G | 5.12 | 4.94E-05 |
|  | Polysaccharide deacetylase | EY04_RS15615 | COG0726 | GM | 4.97 | 5.68E-03 |
|  | Glucan biosynthesis protein D | EY04_RS04860 | cl19308 | - | 2.19 | 7.60E-03 |
|  | Glucose-6-phosphate dehydrogenase | EY04_RS22855 | COG0364 | G | -1.51 | 5.43E-07 |
|  | Sugar ABC transporter ATP-binding protein | EY04_RS21325 | COG1134 | GM | -1.68 | 4.90E-05 |
|  | 2-methylcitrate dehydratase | EY04_RS08815 | COG2079 | G | -2.03 | 4.34E-06 |
|  | Glycerol uptake facilitator GlpF | EY04_RS24265 | COG0580 | G | -2.20 | 1.25E-04 |
|  | MFS transporter | EY04_RS16180 | COG2814 | G | -2.23 | 3.30E-03 |
|  | Sorbosone dehydrogenase | EY04_RS22005 | COG2133 | G | -2.27 | 1.92E-07 |
|  | Sugar ABC transporter permease | EY04_RS21330 | COG1682 | GM | -2.28 | 9.09E-04 |
|  | Glyceraldehyde-3-phosphate dehydrogenase | EY04_RS22920 | COG0057 | G | -3.03 | 3.67E-29 |
|  | Multidrug resistance protein B | EY04_RS19225 | COG2814 | G | -3.31 | 7.21E-05 |
|  | Sugar ABC transporter permease | EY04_RS22885 | COG1175 | G | -3.46 | 2.31E-04 |
|  | DSBA oxidoreductase | EY04_RS00150 | COG2814 | G | -3.60 | 5.14E-03 |
|  | Permease DsdX | EY04_RS22700 | COG2610 | GR | -3.76 | 5.06E-11 |
|  | Gluconokinase | EY04_RS22705 | COG3265 | G | -4.13 | 2.87E-05 |
|  | Sugar ABC transporter ATPase | EY04_RS22875 | COG3839 | G | -4.48 | 6.82E-13 |
|  | Chitin-binding protein | EY04_RS16025 | cl27306 | - | -4.68 | 5.86E-28 |
|  | Sugar ABC transporter substrate-binding protein | EY04_RS22890 | COG1653 | G | -5.68 | 3.12E-27 |
|  | Transporter | EY04_RS10775 | COG0697 | GER | -6.27 | 2.83E-17 |
|  | Chitinase | EY04_RS16020 | COG3469 | G | -7.67 | 5.73E-43 |
|  | Chitin-binding protein | EY04_RS09700 | cl27306 | - | -10.68 | 8.63E-70 |
|  | Chitinase | EY04_RS09705 | COG3469 | G | -10.75 | 1.80E-110 |
| Coenzyme transport and metabolism | Pyridoxine 5'-phosphate synthase | EY04_RS04665 | COG0854 | H | 2.57 | 8.44E-03 |
|  | Monooxygenase | EY04_RS16075 | COG2141 | HR | 1.66 | 8.44E-03 |
|  | Omega amino acid--pyruvate aminotransferase | EY04_RS02980 | COG0161 | H | -1.55 | 2.29E-03 |
|  | Hypothetical protein | EY04_RS17950 | COG1335 | HR | -1.64 | 9.06E-03 |
|  | Poly(3-hydroxyalkanoate) depolymerase | EY04_RS01515 | COG0596 | HR | -1.74 | 1.24E-03 |
|  | Hypothetical protein | EY04_RS29920 | COG0432 | H | -1.97 | 2.41E-05 |
|  | Bifunctional pyrazinamidase/nicotinamidase | EY04_RS16235 | COG1335 | HR | -4.16 | 3.08E-25 |
|  | Hypothetical protein | EY04_RS07950 | COG0161 | H | -4.46 | 3.14E-17 |
|  | MFS transporter | EY04_RS06330 | COG0596 | HR | -6.78 | 3.95E-71 |
| Lipid transport and metabolism | Diaminopimelate decarboxylase | EY04_RS15395 | cl27723 | - | 5.66 | 3.77E-06 |
|  | Acetyl-CoA acetyltransferase | EY04_RS08670 | COG0183 | I | 3.99 | 5.79E-03 |
|  | Pyridine nucleotide-disulfide oxidoreductase | EY04_RS15285 | COG0446 | I | 3.97 | 6.29E-03 |
|  | Acyl-CoA dehydrogenase | EY04_RS27425 | COG1960 | I | 1.86 | 4.77E-03 |
|  | Fatty acid methyltransferase | EY04_RS29115 | COG2230 | I | -1.66 | 1.09E-06 |
|  | Trans-2-enoyl-CoA reductase | EY04_RS12495 | COG3007 | I | -1.66 | 3.39E-05 |
|  | Long-chain fatty acid--CoA ligase | EY04_RS22795 | COG0318 | IQ | -1.99 | 2.53E-07 |
|  | Acetyl-CoA carboxylase subunit alpha | EY04_RS05230 | COG0825 | I | -2.06 | 4.34E-10 |
|  | Acetyl-CoA synthetase | EY04_RS22440 | COG0365 | I | -2.10 | 3.48E-06 |
|  | Acyl-CoA dehydrogenase | EY04_RS08320 | COG1960 | I | -2.40 | 1.58E-08 |
|  | Glycerol acyltransferase | EY04_RS28810 | COG0204 | I | -2.51 | 5.69E-06 |
|  | Poly(R)-hydroxyalkanoic acid synthase | EY04_RS01510 | COG3243 | I | -2.82 | 7.39E-13 |
|  | Poly(R)-hydroxyalkanoic acid synthase | EY04_RS01520 | COG3243 | I | -3.14 | 1.49E-22 |
|  | Glucose-methanol-choline oxidoreductase | EY04_RS15065 | COG2303 | IR | -3.52 | 5.98E-05 |
|  | Poly(3-hydroxyalkanoate) granule-associated protein PhaF | EY04_RS01500 | - | - | -3.52 | 8.85E-26 |
|  | Glycerol acyltransferase | EY04_RS21875 | COG0204 | I | -4.09 | 6.77E-24 |
|  | Dehydratase | EY04_RS27610 | COG2030 | I | -4.31 | 3.86E-26 |
|  | (2Fe-2S)-binding protein HcnB | EY04_RS11545 | COG0446 | I | -5.41 | 1.33E-19 |
|  | Poly(3-hydroxyalkanoate) granule-associated protein PhaI | EY04_RS01495 | - | - | -6.87 | 1.91E-88 |
| Translation, ribosomal structure and biogenesis | Molecular chaperone DnaK | EY04_RS29180 | COG1734 | J | 7.05 | 3.46E-03 |
|  | Threonyl-tRNA synthetase | EY04_RS29130 | COG0441 | J | 4.87 | 2.20E-04 |
|  | tRNA hydroxylase | EY04_RS11880 | cl00264 | - | 2.00 | 1.80E-04 |
|  | 23S rRNA pseudouridylate synthase | EY04_RS08415 | COG0564 | J | 1.78 | 1.96E-03 |
|  | Glucose-1-phosphate cytidylyltransferase | EY04_RS07500 | COG1208 | JM | -1.51 | 2.88E-04 |
|  | Polynucleotide phosphorylase/polyadenylase | EY04_RS03540 | COG1185 | J | -1.69 | 3.80E-04 |
|  | Elongation factor Tu | EY04_RS28215 | COG0050 | J | -1.79 | 1.28E-07 |
|  | Elongation factor Tu | EY04_RS28150 | COG0050 | J | -2.16 | 3.26E-12 |
|  | 50S ribosomal protein L10 | EY04_RS28185 | COG0244 | J | -2.28 | 6.82E-13 |
|  | 50S ribosomal protein L7/L12 | EY04_RS28180 | COG0222 | J | -2.59 | 4.04E-13 |
|  | GNAT family acetyltransferase | EY04_RS06290 | COG1670 | JO | -2.62 | 2.59E-03 |
|  | GTP-binding protein | EY04_RS14290 | COG0480 | J | -2.68 | 5.78E-11 |
|  | Alanyl-tRNA synthetase | EY04_RS08305 | cl26819 | - | -3.45 | 2.91E-06 |
|  | 50S ribosomal protein L21 | EY04_RS16195 | cl09109 | - | -4.88 | 5.63E-04 |
|  | Tryptophan synthase subunit alpha | EY04_RS11035 | cl27535 | - | -5.84 | 5.53E-49 |
|  | Methionyl-tRNA formyltransferase | EY04_RS07955 | cl00395 | - | -7.98 | 8.26E-11 |
| Transcription | GntR family transcriptional regulator | EY04_RS12060 | COG1802 | K | 3.76 | 9.31E-03 |
|  | 2-hydroxyacid dehydrogenase | EY04_RS15680 | COG1396 | K | 3.29 | 8.00E-03 |
|  | RNA polymerase sigma factor | EY04_RS20250 | COG1595 | K | 2.66 | 3.72E-03 |
|  | RNA polymerase subunit sigma-70 | EY04_RS10675 | COG1595 | K | 2.22 | 7.22E-06 |
|  | RNA polymerase sigma factor | EY04_RS00050 | COG1595 | K | 2.19 | 3.90E-04 |
|  | Transcriptional regulator | EY04_RS01770 | cl22854 | - | 2.16 | 6.70E-04 |
|  | Transcriptional regulator | EY04_RS29740 | COG3450 | K | 1.85 | 1.12E-03 |
|  | AraC family transcriptional regulator | EY04_RS04305 | COG2207 | K | 1.81 | 5.14E-03 |
|  | AraC family transcriptional regulator | EY04_RS19450 | COG2207 | K | 1.60 | 9.49E-03 |
|  | GntR family transcriptional regulator | EY04_RS07920 | COG1167 | KE | -1.66 | 2.79E-05 |
|  | H-NS histone MvaT | EY04_RS23865 | - | - | -1.73 | 9.66E-07 |
|  | Cold-shock protein | EY04_RS19040 | COG1278 | K | -1.86 | 2.41E-06 |
|  | AraC family transcriptional regulator | EY04_RS01040 | COG2207 | K | -2.70 | 1.67E-05 |
|  | Transcriptional regulator MvaV | EY04_RS16500 | - | - | -2.92 | 6.27E-16 |
|  | AraC family transcriptional regulator | EY04_RS23115 | COG2207 | K | -2.96 | 4.08E-03 |
|  | GntR family transcriptional regulator | EY04_RS23170 | COG2186 | K | -2.98 | 8.60E-08 |
|  | TetR family transcriptional regulator | EY04_RS01505 | COG1309 | K | -3.20 | 9.55E-05 |
|  | LuxR family transcriptional regulator CsaR | EY04_RS11855 | COG2771 | K | -3.24 | 9.39E-06 |
|  | RNA polymerase sigma factor RpoS | EY04_RS05305 | COG0568 | K | -3.28 | 4.51E-26 |
|  | Cold-shock protein | EY04_RS23025 | COG1278 | K | -3.29 | 3.39E-24 |
|  | Cro/Cl family transcriptional regulator | EY04_RS18125 | COG1396 | K | -3.52 | 1.36E-08 |
|  | PadR family transcriptional regulator | EY04_RS24465 | COG1695 | K | -3.57 | 6.49E-12 |
|  | LysR family transcriptional regulator | EY04_RS27730 | COG0583 | K | -3.78 | 2.79E-04 |
|  | Fis family transcriptional regulator | EY04_RS29535 | cl28069 | - | -4.04 | 5.99E-08 |
|  | Transcriptional regulator PhzR | EY04_RS25710 | COG2771 | R | -4.12 | 1.32E-25 |
|  | Transcriptional regulator | EY04_RS27335 | - | - | -4.13 | 1.71E-14 |
|  | DNA-binding protein | EY04_RS24950 | COG1396 | K | -5.70 | 5.49E-09 |
| Replication, recombination and repair | Cytosine methyltransferase | EY04_RS17080 | COG0270 | L | 2.24 | 2.34E-03 |
|  | Hypothetical protein | EY04_RS29720 | COG2003 | L | -1.54 | 7.44E-03 |
|  | Dipicolinate synthase | EY04_RS04595 | COG0776 | L | -1.62 | 7.56E-06 |
|  | Hypothetical protein | EY04_RS20750 | COG1112 | L | -2.42 | 2.24E-07 |
|  | Chromosome partitioning protein ParA | EY04_RS04995 | - | - | -2.43 | 4.10E-05 |
|  | Competence protein ComEA | EY04_RS21285 | COG1555 | L | -6.06 | 3.82E-06 |
| Cell wall/membrane/envelope biogenesis | N-acetylmuramoyl-L-alanine amidase | EY04_RS29170 | COG0860 | M | 6.31 | 9.49E-08 |
|  | Alanine racemase | EY04_RS29795 | COG0787 | M | 4.64 | 6.41E-04 |
|  | Phospholipase | EY04_RS15425 | COG3511 | M | 3.49 | 3.14E-03 |
|  | Channel protein TolC | EY04_RS18285 | COG1538 | M | 2.26 | 2.34E-03 |
|  | Curli production assembly protein CsgG | EY04_RS18660 | COG1462 | M | 1.70 | 6.80E-04 |
|  | Hemolysin secretion protein D | EY04_RS20255 | COG0845 | MV | 1.50 | 1.35E-03 |
|  | Membrane protein | EY04_RS21290 | COG1086 | MO | -1.59 | 1.11E-04 |
|  | 4-amino-4-deoxy-L-arabinose transferase | EY04_RS14150 | COG1807 | M | -1.64 | 6.90E-03 |
|  | Membrane protein | EY04_RS05755 | COG2885 | M | -1.86 | 2.58E-03 |
|  | Membrane protein | EY04_RS04990 | COG2885 | M | -2.02 | 5.20E-04 |
|  | Porin | EY04_RS22870 | COG3659 | M | -2.11 | 2.96E-09 |
|  | Channel protein TolC | EY04_RS07255 | COG1538 | M | -2.28 | 4.39E-05 |
|  | RND transporter | EY04_RS00160 | COG1538 | M | -2.32 | 6.56E-03 |
|  | Porin | EY04_RS08860 | COG2885 | M | -2.43 | 4.09E-18 |
|  | Multidrug RND transporter | EY04_RS19235 | COG1538 | M | -2.49 | 4.69E-05 |
|  | N-acetylmuramoyl-L-alanine amidase | EY04_RS05345 | COG3023 | M | -2.52 | 1.33E-07 |
|  | Glycosyl transferase | EY04_RS21295 | COG0438 | M | -2.58 | 5.00E-10 |
|  | Secretion protein HlyD | EY04_RS27280 | COG0845 | MV | -2.79 | 2.76E-04 |
|  | Peptidase M50 | EY04_RS00185 | COG0845 | MV | -2.88 | 2.56E-05 |
|  | Acriflavin resistance protein AcrA | EY04_RS17230 | COG0845 | MV | -2.92 | 5.50E-06 |
|  | Hypothetical protein | EY04_RS14305 | COG1520 | M | -3.20 | 1.00E-15 |
|  | Transporter | EY04_RS00220 | COG1538 | M | -4.82 | 1.36E-08 |
| Cell motility | Chemotaxis protein | EY04_RS16315 | COG0840 | NT | 6.21 | 9.78E-06 |
|  | Chemotaxis protein | EY04_RS22555 | COG0840 | NT | 4.04 | 3.91E-03 |
|  | Type III secretion protein ATPase | EY04_RS27165 | COG1157 | NU | 2.61 | 5.04E-03 |
|  | Chemotaxis protein | EY04_RS22955 | COG0840 | NT | 2.60 | 9.45E-04 |
|  | Chemotaxis protein | EY04_RS22505 | COG0840 | NT | 2.59 | 4.19E-05 |
|  | Chemotaxis protein | EY04_RS02170 | COG0840 | NT | 2.48 | 4.35E-04 |
|  | Chemotaxis protein CheW | EY04_RS20840 | COG0835 | NT | 1.62 | 4.90E-03 |
|  | Flagellar hook protein FlgE | EY04_RS22205 | COG1749 | N | -1.64 | 1.26E-05 |
|  | Chemotaxis protein CheY | EY04_RS18880 | cl26036 | - | -1.80 | 8.60E-05 |
| Posttranslational modification, protein turnover, chaperones | Glutathione S-transferase | EY04_RS21630 | COG0625 | O | 5.80 | 1.36E-04 |
|  | Heat-shock protein | EY04_RS09505 | COG0071 | O | 4.78 | 1.48E-09 |
|  | Heat shock protein GrpE | EY04_RS03425 | COG0576 | O | 4.14 | 6.96E-08 |
|  | Heat shock protein 90 | EY04_RS08090 | COG0326 | O | 4.09 | 5.41E-04 |
|  | ATP-dependent protease ATP-binding subunit HslU | EY04_RS01530 | COG1220 | O | 3.93 | 1.13E-07 |
|  | Lon protease | EY04_RS24515 | COG0466 | O | 3.12 | 6.06E-06 |
|  | Protease | EY04_RS29100 | COG0330 | O | 2.87 | 4.22E-03 |
|  | Molecular chaperone DnaK | EY04_RS03430 | COG0443 | O | 2.58 | 5.49E-08 |
|  | Protein disaggregation chaperone | EY04_RS26385 | COG0542 | O | 2.55 | 9.66E-07 |
|  | Molecular chaperone GroES | EY04_RS24130 | COG0234 | O | 2.12 | 4.77E-05 |
|  | Membrane protein | EY04_RS29095 | COG0330 | O | 1.87 | 7.53E-03 |
|  | Molecular chaperone GroEL | EY04_RS24125 | COG0459 | O | 1.78 | 2.71E-04 |
|  | Protein-L-isoaspartate O-methyltransferase | EY04_RS05295 | COG2518 | O | 1.73 | 2.42E-03 |
|  | Clp protease ClpX | EY04_RS19030 | COG0542 | O | 1.63 | 3.21E-06 |
|  | Peptidase S41 | EY04_RS01205 | COG0793 | O | 1.52 | 1.59E-03 |
|  | Glutathione S-transferase | EY04_RS04580 | COG0625 | O | -1.63 | 1.69E-03 |
|  | Peroxidase | EY04_RS13245 | COG2077 | O | -2.23 | 1.53E-03 |
|  | Glutaredoxin | EY04_RS24235 | COG0278 | O | -2.38 | 3.88E-06 |
|  | Hypothetical protein | EY04_RS14340 | COG2234 | O | -3.18 | 1.06E-06 |
|  | ATPase AAA | EY04_RS29530 | COG0542 | O | -4.98 | 3.81E-19 |
| Inorganic ion transport and metabolism | Metal ABC transporter substrate-binding protein | EY04_RS29150 | COG0803 | P | 7.57 | 2.42E-08 |
|  | AcsD protein | EY04_RS15400 | cl27184 | - | 6.35 | 2.61E-11 |
|  | Zinc ABC transporter permease | EY04_RS29155 | COG1108 | P | 6.22 | 2.20E-05 |
|  | Iron ABC transporter substrate-binding protein | EY04_RS02495 | COG0614 | P | 5.84 | 4.19E-05 |
|  | TonB-dependent receptor | EY04_RS24485 | COG1629 | P | 5.55 | 6.75E-12 |
|  | Achromobactin-binding protein | EY04_RS15370 | COG0614 | P | 5.40 | 2.55E-04 |
|  | ABC transporter permease | EY04_RS02490 | COG0609 | P | 5.22 | 4.65E-04 |
|  | ABC transporter substrate-binding protein | EY04_RS11345 | COG0715 | P | 5.16 | 2.86E-03 |
|  | TonB-dependent receptor | EY04_RS15410 | COG1629 | P | 5.02 | 8.62E-38 |
|  | AcsA protein | EY04_RS15375 | cl27184 | - | 5.00 | 3.55E-13 |
|  | TonB-dependent receptor | EY04_RS02505 | COG1629 | P | 4.96 | 8.04E-06 |
|  | ABC transporter substrate-binding protein | EY04_RS30325 | COG0715 | P | 4.93 | 3.72E-03 |
|  | Histidinol phosphatase | EY04_RS02500 | COG1120 | PH | 4.55 | 4.87E-03 |
|  | Iron ABC transporter ATP-binding protein | EY04_RS15355 | COG1120 | PH | 4.31 | 8.69E-03 |
|  | Siderophore biosynthesis protein SbnG | EY04_RS15380 | cl21481 | - | 4.28 | 1.54E-03 |
|  | ABC transporter permease | EY04_RS00445 | COG1613 | P | 4.24 | 8.36E-08 |
|  | AcsC protein | EY04_RS15385 | cl27184 | - | 3.77 | 1.41E-09 |
|  | Siderophore ABC transporter permease | EY04_RS15365 | COG0609 | P | 3.26 | 8.44E-03 |
|  | Peptide ABC transporter substrate-binding protein | EY04_RS00045 | COG3712 | PT | 3.03 | 2.50E-07 |
|  | admium ABC transporter ATPase | EY04_RS29110 | COG2217 | P | 2.83 | 1.49E-05 |
|  | TonB-dependent receptor | EY04_RS10685 | COG1629 | P | 2.69 | 5.19E-05 |
|  | TonB-dependent receptor | EY04_RS22940 | COG1629 | P | 2.45 | 6.05E-11 |
|  | Sulfite reductase | EY04_RS18485 | COG0155 | P | 1.90 | 9.07E-04 |
|  | Iron dicitrate transport regulator FecR | EY04_RS17435 | COG3712 | PT | 1.73 | 1.25E-04 |
|  | Adenylylsulfate kinase | EY04_RS04025 | COG2895 | P | 1.62 | 6.26E-03 |
|  | Cystathionine gamma-synthase | EY04_RS30070 | COG0672 | P | 1.55 | 4.88E-04 |
|  | Catalase | EY04_RS28010 | COG0753 | P | -1.84 | 5.43E-04 |
|  | Carbonate dehydratase | EY04_RS28575 | COG0288 | P | -2.06 | 3.21E-05 |
|  | ABC transporter substrate-binding protein | EY04_RS21760 | COG1840 | P | -2.21 | 3.55E-05 |
|  | copper resistance protein CopZ | EY04_RS02835 | COG2608 | P | -2.38 | 7.18E-03 |
|  | Ferritin | EY04_RS23755 | COG0783 | PV | -2.54 | 7.23E-11 |
|  | FAD-binding protein | EY04_RS24470 | COG2375 | P | -2.74 | 3.19E-10 |
|  | Sulfite reductase | EY04_RS13220 | COG0155 | P | -3.25 | 1.33E-23 |
|  | Adenylylsulfate kinase | EY04_RS00225 | COG0529 | P | -3.54 | 3.21E-06 |
|  | Catalase | EY04_RS26655 | COG0753 | P | -3.59 | 6.29E-04 |
|  | Metal ABC transporter ATPase | EY04_RS02825 | COG2217 | P | -3.93 | 5.45E-16 |
|  | Potassium transporter | EY04_RS17625 | COG0475 | P | -4.42 | 1.09E-10 |
| Secondary metabolites biosynthesis, transport and catabolism | Acyl-homoserine lactone acylase subunit beta | EY04_RS13450 | COG2366 | Q | 1.79 | 2.09E-06 |
|  | Dienelactone hydrolase | EY04_RS22180 | COG0412 | Q | -2.26 | 6.92E-04 |
|  | Tryptophan halogenase PrnA | EY04_RS17650 | cl26176 | - | -4.17 | 1.52E-29 |
|  | (2Fe-2S)-binding protein HcnA | EY04_RS11540 | cl09928 | - | -4.31 | 1.65E-12 |
|  | Serine 3-dehydrogenase | EY04_RS11085 | COG2931 | Q | -4.59 | 2.33E-46 |
|  | Inducer of phenazine B | EY04_RS07930 | - | - | -4.77 | 3.91E-28 |
|  | Inducer of phenazine A | EY04_RS07925 | - | - | -5.23 | 3.92E-36 |
|  | PrnB | EY04_RS17645 | - | - | -5.61 | 6.01E-18 |
|  | FAD-dependent oxidoreductase PrnC | EY04_RS17640 | cl27554 | - | -5.92 | 2.07E-59 |
|  | Serine 3-dehydrogenase | EY04_RS15795 | COG2931 | Q | -6.39 | 1.71E-60 |
|  | 2Fe-2S ferredoxin PrnD | EY04_RS17635 | cl28556 | - | -6.64 | 3.57E-20 |
|  | Ring-cleavage extradiol dioxygenase | EY04_RS14300 | COG0346 | Q | -7.22 | 2.61E-17 |
|  | Anthranilate synthase PhzE | EY04_RS25735 | cl27696 | - | -7.24 | 5.72E-61 |
|  | Phospho-2-dehydro-3-deoxyheptonate aldolase PhzC | EY04_RS25725 | cl03230 | - | -8.25 | 1.79E-60 |
|  | Phenazine biosynthesis protein PhzO | EY04_RS25750 | cl26441 | - | -8.63 | 4.50E-139 |
|  | Phenazine biosynthesis protein PhzG | EY04_RS25745 | cl25685 | - | -8.71 | 7.91E-27 |
|  | Isochorismatase | EY04_RS25730 | COG1535 | Q | -8.92 | 2.22E-17 |
|  | Phenazine biosynthesis protein PhzA | EY04_RS25715 | cl09109 | - | -10.14 | 6.46E-23 |
|  | Phenazine biosynthesis protein PhzB | EY04_RS25720 | - | - | -10.68 | 8.32E-17 |
|  | 2,3-dihydro-3-hydroxyanthranilate isomerase | EY04_RS25740 | COG0384 | R | -8.34 | 7.81E-29 |
| General function prediction only | Hypothetical protein | EY04_RS29185 | COG0523 | R | 7.37 | 9.78E-08 |
|  | Carbonate dehydratase | EY04_RS29140 | COG0663 | R | 7.03 | 3.71E-07 |
|  | Carbon-nitrogen hydrolase | EY04_RS03865 | COG0388 | R | 3.14 | 1.61E-05 |
|  | Cobalamin biosynthesis protein CobW | EY04_RS18435 | COG0523 | R | 1.89 | 9.49E-03 |
|  | Protease TldD | EY04_RS03870 | COG0312 | R | 1.80 | 3.49E-03 |
|  | Cytochrome D ubiquinol oxidase subunit II | EY04_RS05545 | COG1611 | R | -1.84 | 8.96E-09 |
|  | NADP-dependent oxidoreductase | EY04_RS16465 | COG0667 | R | -2.19 | 1.45E-04 |
|  | Carboxylesterase | EY04_RS04520 | COG0400 | R | -2.22 | 1.25E-03 |
|  | ABC transporter | EY04_RS14320 | COG4586 | R | -2.36 | 4.74E-04 |
|  | Arylsulfate sulfotransferase | EY04_RS06145 | COG4321 | R | -2.84 | 5.93E-04 |
|  | Hypothetical protein | EY04_RS30945 | COG2353 | R | -2.94 | 6.11E-21 |
| Function unknown | GTP cyclohydrolase | EY04_RS29165 | cl00642 | - | 8.07 | 4.80E-06 |
|  | Hypothetical protein | EY04_RS00955 | - | - | 7.73 | 3.29E-08 |
|  | Manganese ABC transporter ATP-binding protein | EY04_RS29160 | cl28181 | - | 6.56 | 5.35E-06 |
|  | Hypothetical protein | EY04_RS29190 | - | - | 6.47 | 4.54E-06 |
|  | Cobalamin biosynthesis protein CobW | EY04_RS29195 | cl26870 | - | 5.16 | 5.62E-04 |
|  | Nickel uptake transporter family protein | EY04_RS15510 | - | - | 5.13 | 6.41E-04 |
|  | Glutamine synthetase | EY04_RS29175 | - | - | 4.98 | 9.24E-05 |
|  | S-adenosylmethionine: 2-demethylmenaquinone methyltransferase | EY04_RS15345 | cl00480 | - | 4.57 | 5.49E-03 |
|  | MFS transporter | EY04_RS29750 | - | - | 4.42 | 6.30E-03 |
|  | Membrane protein | EY04_RS15505 | cl21495 | - | 4.36 | 1.19E-03 |
|  | ABC transporter substrate-binding protein | EY04_RS26860 | cl26593 | - | 4.24 | 2.58E-03 |
|  | Filamentous hemagglutinin | EY04_RS20950 | - | - | 4.17 | 4.09E-26 |
|  | Hypothetical protein | EY04_RS08720 | - | - | 4.17 | 2.80E-03 |
|  | Hypothetical protein | EY04_RS27905 | - | - | 4.16 | 3.54E-04 |
|  | Hypothetical protein | EY04_RS16570 | - | - | 2.91 | 2.15E-04 |
|  | Hemolysin D | EY04_RS18300 | cl25633 | - | 2.73 | 4.65E-12 |
|  | MFS transporter | EY04_RS01165 | - | - | 2.68 | 2.78E-08 |
|  | Hypothetical protein | EY04_RS21595 | - | - | 2.42 | 2.56E-03 |
|  | Hypothetical protein | EY04_RS27900 | - | - | 2.39 | 5.14E-03 |
|  | Hypothetical protein | EY04_RS06970 | - | - | 2.36 | 2.36E-04 |
|  | Hypothetical protein | EY04_RS05895 | - | - | 2.28 | 1.53E-03 |
|  | GDSL family lipase | EY04_RS06315 | cl28698 | - | 2.23 | 6.41E-04 |
|  | Trotein-disulfide isomerase | EY04_RS00965 | cl00388 | - | 2.16 | 6.35E-04 |
|  | TonB-dependent receptor | EY04_RS00960 | cl25796 | - | 2.09 | 1.52E-03 |
|  | Hypothetical protein | EY04_RS24745 | cl10470 | - | 1.96 | 9.47E-04 |
|  | Peptide ABC transporter substrate-binding protein | EY04_RS19760 | cl27113 | - | 1.95 | 5.54E-03 |
|  | Hypothetical protein | EY04_RS31335 | - | - | 1.87 | 5.65E-03 |
|  | Membrane protein | EY04_RS04135 | cl00596 | - | 1.72 | 8.96E-03 |
|  | Adenylate cyclase | EY04_RS29975 | cl27585 | - | 1.69 | 8.13E-03 |
|  | AP endonuclease | EY04_RS16290 | - | - | 1.63 | 6.38E-04 |
|  | Hypothetical protein | EY04_RS08660 | - | - | 1.60 | 6.41E-04 |
|  | Cytotoxin | EY04_RS18290 | - | - | 1.58 | 5.13E-07 |
|  | Serine protease | EY04_RS19790 | cl27557 | - | -1.57 | 2.46E-04 |
|  | Glucokinase | EY04_RS22910 | cl17037 | - | -1.58 | 1.47E-04 |
|  | Oxidase | EY04_RS12010 | - | - | -1.60 | 4.61E-03 |
|  | Hypothetical protein | EY04_RS26930 | - | - | -1.66 | 5.72E-03 |
|  | Hypothetical protein | EY04_RS21360 | - | - | -1.67 | 1.01E-03 |
|  | Site-specific recombinase | EY04_14965 | - | - | -1.68 | 8.85E-06 |
|  | Integrase | EY04_RS27380 | - | - | -1.69 | 8.42E-04 |
|  | Membrane protein | EY04_RS15430 | - | - | -1.69 | 3.44E-04 |
|  | Hypothetical protein | EY04_RS15915 | - | - | -1.69 | 2.93E-04 |
|  | Hypothetical protein | EY04_RS00170 | - | - | -1.71 | 4.66E-03 |
|  | Phosphatidylcholine-hydrolyzing phospholipase | EY04_RS03775 | - | - | -1.72 | 4.61E-03 |
|  | Hypothetical protein | EY04_RS05455 | cl26558 | - | -1.76 | 5.80E-03 |
|  | Hypothetical protein | EY04_RS27490 | - | - | -1.82 | 4.11E-07 |
|  | Hypothetical protein | EY04_RS14680 | cl20185 | - | -1.84 | 1.02E-07 |
|  | Hypothetical protein | EY04_RS19770 | - | - | -1.88 | 7.53E-06 |
|  | Hypothetical protein | EY04_RS27210 | - | - | -1.96 | 6.27E-07 |
|  | ATPase | EY04_RS30435 | - | - | -1.98 | 5.55E-04 |
|  | Hypothetical protein | EY04_RS25350 | - | - | -2.02 | 1.14E-05 |
|  | Hypothetical protein | EY04_RS28565 | - | - | -2.03 | 7.63E-05 |
|  | Membrane protein | EY04_RS09710 | cl01427 | - | -2.06 | 5.65E-03 |
|  | Hypothetical protein | EY04_RS06490 | - | - | -2.06 | 1.79E-07 |
|  | Hypothetical protein | EY04_RS23405 | - | - | -2.08 | 4.89E-07 |
|  | Hypothetical protein | EY04_RS05065 | - | - | -2.13 | 4.36E-04 |
|  | Azurin | EY04_RS02270 | cl19115 | - | -2.15 | 4.82E-09 |
|  | Hypothetical protein | EY04_RS17800 | - | - | -2.22 | 4.66E-03 |
|  | Hypothetical protein | EY04_RS25355 | - | - | -2.23 | 1.08E-07 |
|  | Hypothetical protein | EY04_RS29455 | cl27250 | - | -2.26 | 2.12E-07 |
|  | Hypothetical protein | EY04_RS07275 | cl17362 | - | -2.27 | 5.88E-03 |
|  | Acetyltransferase | EY04_RS06715 | - | - | -2.31 | 1.94E-05 |
|  | Hypothetical protein | EY04_RS23045 | cl26002 | - | -2.31 | 7.53E-07 |
|  | Peptidoglycan-binding protein LysM | EY04_RS00895 | COG1652 | S | -2.31 | 1.91E-09 |
|  | Cyclic nucleotide-binding protein | EY04_RS31405 | cl15354 | - | -2.38 | 5.08E-04 |
|  | Hypothetical protein | EY04_RS15495 | cl01480 | - | -2.42 | 1.34E-06 |
|  | Hypothetical protein | EY04_RS13235 | - | - | -2.46 | 1.50E-08 |
|  | Hypothetical protein | EY04_RS20865 | - | - | -2.47 | 9.18E-04 |
|  | Hypothetical protein | EY04_RS13695 | - | - | -2.48 | 3.30E-07 |
|  | Hypothetical protein | EY04_RS09395 | - | - | -2.49 | 6.21E-07 |
|  | Hypothetical protein | EY04_RS09990 | - | - | -2.50 | 2.20E-06 |
|  | Hypothetical protein | EY04_RS14315 | - | - | -2.51 | 2.48E-05 |
|  | 3-oxoacyl-ACP synthase | EY04_RS20365 | cl28397 | - | -2.52 | 2.38E-03 |
|  | Hypothetical protein | EY04_RS29845 | - | - | -2.52 | 6.54E-05 |
|  | Hypothetical protein | EY04_RS15950 | - | - | -2.53 | 2.44E-04 |
|  | Endonuclease | EY04_RS19870 | cl28452 | - | -2.54 | 2.00E-06 |
|  | Hypothetical protein | EY04_RS23590 | - | - | -2.59 | 5.26E-03 |
|  | Multidrug transporter | EY04_RS00180 | cl25633 | - | -2.59 | 3.84E-04 |
|  | Hypothetical protein | EY04_RS05620 | - | - | -2.60 | 6.30E-03 |
|  | Spore coat protein | EY04_RS19385 | cl02253 | - | -2.65 | 4.00E-03 |
|  | Hypothetical protein | EY04_RS20020 | - | - | -2.67 | 6.35E-04 |
|  | Hypothetical protein | EY04_21780 | - | - | -2.69 | 4.96E-11 |
|  | Hypothetical protein | EY04_RS13225 | cl01526 | - | -2.70 | 2.55E-08 |
|  | Membrane protein | EY04_RS24955 | COG1738 | S | -2.73 | 1.44E-09 |
|  | Hypothetical protein | EY04_RS01075 | - | - | -2.75 | 1.94E-06 |
|  | Hypothetical protein | EY04_07220 | - | - | -2.78 | 1.91E-09 |
|  | Hypothetical protein | EY04_RS09995 | - | - | -2.78 | 1.46E-04 |
|  | Hypothetical protein | EY04_RS10000 | - | - | -2.79 | 6.79E-05 |
|  | Hypothetical protein | EY04_21785 | - | - | -2.91 | 1.91E-09 |
|  | Hypothetical protein | EY04_18755 | - | - | -2.95 | 1.14E-06 |
|  | Hypothetical protein | EY04_RS19325 | - | - | -2.96 | 1.80E-04 |
|  | Hypothetical protein | EY04_RS18360 | - | - | -2.96 | 3.38E-09 |
|  | Hypothetical protein | EY04_RS05400 | - | - | -2.97 | 1.35E-03 |
|  | Hypothetical protein | EY04_RS26995 | - | - | -2.99 | 2.77E-03 |
|  | Hypothetical protein | EY04_RS05515 | - | - | -3.00 | 3.72E-03 |
|  | Hypothetical protein | EY04_RS05465 | cl15796 | - | -3.00 | 4.58E-03 |
|  | Hypothetical protein | EY04_RS19775 | - | - | -3.02 | 1.86E-09 |
|  | Hypothetical protein | EY04_RS19990 | - | - | -3.03 | 2.39E-04 |
|  | Serine protease | EY04_RS15765 | cl22877 | - | -3.06 | 1.51E-07 |
|  | Hypothetical protein | EY04_RS23050 | - | - | -3.07 | 1.49E-08 |
|  | Phospholipid-binding protein | EY04_RS26225 | cl27094 | - | -3.08 | 2.04E-18 |
|  | Hypothetical protein | EY04_RS15070 | - | - | -3.10 | 6.79E-05 |
|  | Hypothetical protein | EY04_RS10230 | cl00303 | - | -3.10 | 6.49E-04 |
|  | Hypothetical protein | EY04_RS01045 | - | - | -3.26 | 3.04E-13 |
|  | Glycosyl hydrolase | EY04_RS00215 | - | - | -3.27 | 1.69E-17 |
|  | Hypothetical protein | EY04_RS08715 | - | - | -3.27 | 8.07E-04 |
|  | Hypothetical protein | EY04_RS28670 | cl01888 | - | -3.29 | 1.21E-06 |
|  | Hypothetical protein | EY04_RS18415 | - | - | -3.31 | 6.02E-05 |
|  | Hypothetical protein | EY04_RS06285 | - | - | -3.32 | 8.84E-22 |
|  | Ion channel protein Tsx | EY04_RS10235 | cl04114 | - | -3.33 | 2.18E-03 |
|  | Hypothetical protein | EY04_RS12635 | - | - | -3.34 | 1.21E-03 |
|  | Hypothetical protein | EY04_RS13205 | - | - | -3.38 | 9.69E-06 |
|  | Hypothetical protein | EY04_RS10060 | - | - | -3.39 | 1.66E-18 |
|  | Hypothetical protein | EY04_RS10785 | - | - | -3.40 | 4.31E-07 |
|  | Hypothetical protein | EY04_RS00395 | - | - | -3.42 | 7.30E-03 |
|  | Hypothetical protein | EY04_RS18395 | - | - | -3.52 | 7.63E-05 |
|  | Hypothetical protein | EY04_RS05500 | - | - | -3.54 | 5.68E-03 |
|  | Hypothetical protein | EY04_RS18385 | cl25370 | - | -3.61 | 1.90E-10 |
|  | Hypothetical protein | EY04_RS12375 | - | - | -3.66 | 2.46E-07 |
|  | Hypothetical protein | EY04_RS11535 | - | - | -3.71 | 1.02E-06 |
|  | Sugar deacetylase | EY04_RS14310 | cl28093 | - | -3.72 | 8.74E-10 |
|  | Hypothetical protein | EY04_RS07130 | - | - | -3.77 | 1.96E-11 |
|  | Hypothetical protein | EY04_RS20030 | - | - | -3.79 | 3.58E-06 |
|  | Hypothetical protein | EY04_RS25960 | - | - | -3.82 | 5.49E-22 |
|  | Prevent-host-death protein | EY04_RS01320 | cl09153 | - | -3.84 | 4.48E-03 |
|  | Hypothetical protein | EY04_RS00845 | - | - | -3.84 | 9.83E-05 |
|  | Hypothetical protein | EY04_RS17535 | - | - | -4.00 | 2.66E-19 |
|  | Porin | EY04_RS23775 | - | - | -4.09 | 1.12E-42 |
|  | C4-dicarboxylate ABC transporter | EY04_RS21880 | cl04176 | - | -4.10 | 1.95E-30 |
|  | Hypothetical protein | EY04_RS26560 | - | - | -4.13 | 2.55E-24 |
|  | Hypothetical protein | EY04_RS01050 | - | - | -4.16 | 4.10E-33 |
|  | Methyltransferase | EY04_RS14345 | cl28097 | - | -4.17 | 6.15E-11 |
|  | Fusaric acid resistance protein | EY04_RS07970 | COG1289 | S | -4.18 | 1.44E-05 |
|  | Amidohydrolase | EY04_RS20460 | cl26822 | - | -4.21 | 1.40E-13 |
|  | Hypothetical protein | EY04_RS20040 | - | - | -4.28 | 2.82E-33 |
|  | Hypothetical protein | EY04_RS11390 | - | - | -4.28 | 2.53E-07 |
|  | Hypothetical protein | EY04_RS04575 | - | - | -4.29 | 1.41E-06 |
|  | Acetyltransferase | EY04_RS00190 | cl26092 | - | -4.33 | 4.03E-06 |
|  | Nuclease | EY04_01095 | - | - | -4.39 | 2.02E-08 |
|  | Hypothetical protein | EY04_RS14665 | - | - | -4.41 | 5.49E-32 |
|  | Hypothetical protein | EY04_RS08300 | - | - | -4.45 | 5.44E-08 |
|  | Hypothetical protein | EY04_RS08540 | - | - | -4.58 | 9.78E-07 |
|  | Hypothetical protein | EY04_RS05385 | - | - | -4.63 | 5.76E-03 |
|  | Hypothetical protein | EY04_RS14655 | - | - | -4.64 | 3.58E-05 |
|  | Hypothetical protein | EY04_RS05435 | - | - | -4.71 | 3.80E-04 |
|  | Hypothetical protein | EY04_RS00195 | - | - | -4.73 | 1.40E-04 |
|  | Hypothetical protein | EY04_RS05505 | - | - | -4.80 | 1.92E-03 |
|  | Hypothetical protein | EY04_RS13340 | - | - | -4.83 | 2.72E-17 |
|  | Phosphatidylserine decarboxylase | EY04_RS10850 | cl03656 | - | -4.88 | 1.03E-24 |
|  | Hypothetical protein | EY04_RS11560 | - | - | -4.91 | 1.22E-37 |
|  | Hypothetical protein | EY04_RS14330 | cl21474 | - | -4.92 | 4.75E-05 |
|  | Hypothetical protein | EY04_RS07945 | cl27640 | - | -4.95 | 7.56E-32 |
|  | Sugar isomerase | EY04_RS22895 | cl23840 | - | -5.01 | 2.51E-12 |
|  | ABC transporter permease | EY04_RS14325 | cl21474 | - | -5.04 | 2.53E-03 |
|  | Hypothetical protein | EY04_RS31325 | - | - | -5.10 | 3.77E-14 |
|  | Hypothetical protein | EY04_RS10670 | - | - | -5.10 | 3.09E-04 |
|  | Dockerin | EY04_RS05335 | - | - | -5.15 | 1.07E-27 |
|  | Hypothetical protein | EY04_17910 | - | - | -5.17 | 5.08E-48 |
|  | Hypothetical protein | EY04_12710 | cl11966 | - | -5.22 | 3.67E-23 |
|  | Mercuric reductase | EY04_07515 | - | - | -5.36 | 8.47E-06 |
|  | Leucyl aminopeptidase (aminopeptidase T) | EY04_RS07410 | - | - | -5.38 | 4.22E-24 |
|  | Hypothetical protein | EY04_RS13890 | - | - | -5.43 | 5.60E-27 |
|  | Inosamine-phosphate amidinotransferase 1 | EY04_RS07935 | cl19186 | - | -5.53 | 1.49E-18 |
|  | Lipoprotein | EY04_RS13885 | - | - | -5.62 | 1.58E-37 |
|  | Membrane protein | EY04_RS20045 | - | - | -5.63 | 7.46E-34 |
|  | Hypothetical protein | EY04_RS24450 | - | - | -5.71 | 5.38E-26 |
|  | Hypothetical protein | EY04_RS23175 | - | - | -5.72 | 6.97E-28 |
|  | dialkylrecorsinol condensing enzyme | EY04_RS20370 | - | - | -5.81 | 1.07E-03 |
|  | Hypothetical protein | EY04_RS09900 | - | - | -5.83 | 7.63E-05 |
|  | Hypothetical protein | EY04_RS01055 | - | - | -5.87 | 2.26E-27 |
|  | Hypothetical protein | EY04_RS00390 | - | - | -5.96 | 4.46E-08 |
|  | Hypothetical protein | EY04_RS24445 | - | - | -6.01 | 1.46E-28 |
|  | Sulfotransferase | EY04_RS00210 | - | - | -6.07 | 1.68E-08 |
|  | Hypothetical protein | EY04_RS30255 | - | - | -6.08 | 2.01E-05 |
|  | Type VI secretion protein | EY04_RS29540 | - | - | -6.08 | 3.70E-04 |
|  | Hypothetical protein | EY04_RS16685 | - | - | -6.11 | 1.38E-21 |
|  | R body protein RebB-like protein | EY04_RS00400 | - | - | -6.33 | 2.59E-19 |
|  | Signal peptide protein | EY04_RS29545 | cl00062 | - | -6.53 | 8.86E-11 |
|  | Hypothetical protein | EY04_RS29515 | - | - | -6.59 | 9.66E-07 |
|  | Hypothetical protein | EY04_03130 | - | - | -6.88 | 3.65E-50 |
|  | Hypothetical protein | EY04_32005 | - | - | -6.91 | 1.27E-07 |
|  | Hypothetical protein | EY04_RS23185 | - | - | -7.00 | 1.07E-47 |
|  | Hypothetical protein | EY04_RS17125 | - | - | -7.23 | 2.11E-62 |
|  | Hypothetical protein | EY04_RS23180 | - | - | -7.30 | 1.28E-20 |
|  | Hypothetical protein | EY04_RS29505 | - | - | -7.47 | 2.82E-09 |
|  | Hypothetical protein | EY04_RS12155 | - | - | -7.48 | 1.13E-11 |
|  | Hypothetical protein | EY04_RS14295 | - | - | -7.69 | 7.59E-27 |
|  | Type VI secretion protein | EY04_RS29490 | cl01402 | - | -7.79 | 3.45E-35 |
|  | R body protein RebB-like protein | EY04_RS00405 | - | - | -7.82 | 1.08E-23 |
|  | Hypothetical protein | EY04_RS29510 | - | - | -7.82 | 2.42E-23 |
|  | Hypothetical protein | EY04_RS23190 | - | - | -8.31 | 6.52E-151 |
|  | Hypothetical protein | EY04_RS14645 | - | - | -10.87 | 3.36E-120 |
| Signal transduction mechanisms | Iron dicitrate transport regulator FecR | EY04_RS10680 | cl27113 | - | 2.92 | 5.38E-13 |
|  | Diguanylate cyclase | EY04_RS31485 | COG2199 | T | 2.87 | 9.85E-03 |
|  | Histidine kinase | EY04_RS27430 | COG0642 | T | 2.73 | 1.98E-06 |
|  | Diguanylate phosphodiesterase | EY04_RS15460 | COG2200 | T | 1.97 | 1.34E-06 |
|  | Universal stress protein | EY04_RS15625 | COG0589 | T | 1.96 | 7.65E-03 |
|  | Histidine kinase | EY04_RS18280 | COG0642 | T | 1.71 | 8.76E-04 |
|  | Chemotaxis protein CheY | EY04_RS29425 | COG0784 | T | 1.64 | 8.74E-03 |
|  | Hypothetical protein | EY04_RS03635 | COG0790 | T | 1.64 | 1.41E-04 |
|  | Histidine kinase | EY04_RS02065 | - | - | 1.64 | 8.15E-03 |
|  | Protein kinase | EY04_RS11790 | COG0515 | T | -1.55 | 3.91E-03 |
|  | Crp/Fnr family transcriptional regulator | EY04_RS06720 | COG0664 | T | -1.70 | 5.63E-03 |
|  | LuxR family transcriptional regulator | EY04_RS02735 | COG2197 | TK | -1.71 | 2.03E-04 |
|  | LuxR family transcriptional regulator | EY04_RS23625 | COG2197 | TK | -1.77 | 5.88E-06 |
|  | Anti-anti-sigma factor | EY04_RS07650 | COG1366 | T | -2.37 | 2.55E-07 |
|  | Histidine kinase | EY04_RS20085 | COG0642 | T | -2.63 | 4.77E-04 |
|  | Diguanylate cyclase | EY04_RS18890 | COG2200 | T | -2.64 | 8.12E-06 |
|  | Diguanylate cyclase | EY04_RS18885 | cl25447 | - | -3.13 | 1.23E-11 |
|  | Protein phosphatase | EY04_RS29570 | COG0631 | T | -3.23 | 9.55E-03 |
|  | Chemotaxis protein CheY | EY04_RS07655 | COG0745 | TK | -3.27 | 1.70E-17 |
|  | Histidine kinase | EY04_RS18900 | cl27674 | - | -3.35 | 1.79E-04 |
|  | Histidine kinase | EY04_RS02775 | cl26036 | - | -4.22 | 2.53E-07 |
|  | LuxR family transcriptional regulator | EY04_RS06740 | COG2197 | TK | -4.47 | 9.63E-06 |
|  | Diguanylate cyclase | EY04_RS31065 | cl25447 | - | -4.96 | 2.29E-26 |
|  | Hypothetical protein | EY04_RS29585 | COG0790 | T | -5.20 | 2.50E-09 |
|  | Acyl-homoserine-lactone synthase PhzI | EY04_RS25705 | cl17182 | - | -5.53 | 1.51E-48 |
|  | Serine/threonine protein kinase | EY04_RS29575 | COG0515 | T | -5.67 | 1.52E-04 |
|  | Acyl-homoserine-lactone synthase CsaI | EY04_RS11850 | cl17182 | - | -6.06 | 2.63E-13 |
| Intracellular trafficking, secretion, and vesicular transport | ShlB family hemolysin secretion/activation protein | EY04_RS20945 | COG2831 | U | 2.89 | 3.94E-03 |
|  | Preprotein translocase subunit YajC | EY04_RS24760 | COG1862 | U | -1.71 | 2.53E-03 |
|  | Secretin | EY04_RS23040 | COG4964 | UW | -2.14 | 2.28E-08 |
|  | Type VI secretion protein | EY04_RS29555 | COG3522 | U | -2.32 | 1.79E-03 |
|  | Type VI secretion protein | EY04_RS29520 | COG3519 | U | -2.62 | 1.39E-04 |
|  | Pilus assembly protein PilA | EY04_RS23110 | COG3847 | UW | -2.85 | 3.28E-03 |
|  | Preprotein translocase subunit SecB | EY04_RS01230 | COG1952 | U | -3.08 | 1.90E-07 |
|  | Type IV secretion protein Rhs | EY04_RS23410 | COG3501 | UXR | -3.09 | 1.92E-09 |
|  | Type VI secretion protein | EY04_RS29525 | COG3520 | U | -3.13 | 7.18E-04 |
|  | Hypothetical protein | EY04_RS20025 | COG4104 | U | -3.40 | 6.39E-05 |
|  | Hypothetical protein | EY04_RS01085 | COG4104 | U | -3.55 | 5.56E-03 |
|  | EvpB family type VI secretion protein | EY04_RS17870 | COG3517 | U | -3.83 | 4.10E-05 |
|  | Type IV secretion protein Rhs | EY04_RS00555 | COG3501 | UXR | -4.18 | 1.97E-09 |
|  | Type IV secretion protein Rhs | EY04_RS20050 | COG3501 | UXR | -4.41 | 2.06E-42 |
|  | Type VI secretion protein | EY04_RS29550 | COG3521 | U | -4.59 | 7.86E-04 |
|  | Membrane protein | EY04_RS29560 | COG3455 | U | -4.96 | 1.73E-09 |
|  | Type VI secretion protein VasK | EY04_RS29565 | COG3523 | U | -5.00 | 1.46E-25 |
|  | Type IV secretion protein Rhs | EY04_RS29580 | COG3501 | UXR | -5.35 | 1.09E-10 |
|  | Type VI secretion protein ImpA | EY04_RS29485 | COG3515 | U | -5.65 | 4.67E-15 |
|  | Hypothetical protein | EY04_RS29500 | COG3518 | U | -6.08 | 7.23E-13 |
|  | Type VI secretion protein | EY04_RS29495 | COG3517 | U | -6.11 | 1.03E-69 |
|  | Hypothetical protein | EY04_RS00560 | COG3157 | U | -7.00 | 1.79E-47 |
|  | Pilus assembly protein | EY04_RS02770 | COG3847 | UW | -7.19 | 1.35E-13 |
| Defense mechanisms | Peroxidase | EY04_RS30315 | COG0450 | V | 2.69 | 6.41E-04 |
|  | Peptidase C39 | EY04_RS18295 | COG2274 | V | 2.33 | 1.20E-08 |
|  | Peptidase C39 | EY04_RS18305 | COG2274 | V | 1.98 | 6.69E-07 |
|  | Transporter | EY04_RS16090 | COG0841 | V | 1.84 | 4.95E-03 |
|  | Peroxidase | EY04_RS10015 | COG0450 | V | -1.81 | 4.62E-04 |
|  | Transporter | EY04_RS17225 | COG0841 | V | -2.39 | 4.99E-06 |
|  | Peroxiredoxin OsmC | EY04_RS28650 | COG1764 | V | -2.54 | 8.95E-07 |
|  | ATP-binding protein | EY04_RS27285 | COG2274 | V | -2.64 | 6.07E-03 |
|  | Alkyl hydroperoxide reductase | EY04_RS16520 | COG0450 | V | -2.95 | 2.74E-09 |
|  | Hemolysin D | EY04_RS19230 | COG1566 | V | -3.60 | 2.15E-07 |
|  | Pyocin R2, holin | EY04_RS05340 | - | - | -4.42 | 5.76E-06 |
| Extracellular structures | Molecular chaperone | EY04_RS19375 | COG3121 | W | -1.83 | 4.54E-03 |
| Mobilome: prophages, transposons | Phage tail protein | EY04_RS05380 | - | - | -2.37 | 8.85E-03 |
|  | Baseplate J protein | EY04_RS05365 | cl01294 | - | -2.63 | 1.61E-04 |
|  | Phage tail protein | EY04_RS05395 | cl01390 | - | -2.71 | 2.93E-04 |
|  | Tail protein | EY04_RS05410 | - | - | -2.96 | 9.78E-06 |
|  | Tail sheath protein | EY04_RS05440 | cl26913 | - | -3.23 | 2.79E-15 |
|  | Tail protein | EY04_RS05390 | COG3497 | X | -3.29 | 2.82E-10 |
|  | Tail protein | EY04_RS05375 | cl26341 | - | -3.49 | 1.97E-09 |
|  | Phage tail protein | EY04_RS05445 | - | - | -3.86 | 3.99E-04 |
|  | Baseplate assembly protein | EY04_RS05355 | cl17812 | - | -3.96 | 1.10E-05 |
|  | Tail protein | EY04_RS00200 | cl26890 | - | -4.50 | 5.76E-06 |
|  | Caudovirales tail fiber assembly protein | EY04_RS05330 | - | - | -4.56 | 3.41E-24 |
|  | sRNA | *rsmZ* |  |  | -6.57 | 5.06E-08 |
